# Revealing a distortive polar order buried in the Fermi sea

**DOI:** 10.1126/sciadv.adn0929

**Published:** 2024-07-12

**Authors:** Jiaojian Shi, Wenjing You, Xian Li, Frank Y. Gao, Xinyue Peng, Shangjie Zhang, Jiangxu Li, Yang Zhang, Liang Fu, Patrick J. Taylor, Keith A. Nelson, Edoardo Baldini

**Affiliations:** ^1^ Department of Materials Science and Engineering, Stanford University, Stanford, CA 94305, USA.; ^2^ Department of Physics and Center for Complex Quantum Systems, The University of Texas at Austin, Austin, TX 78712, USA.; ^3^ Department of Chemistry, Massachusetts Institute of Technology, Cambridge, MA 02139, USA.; ^4^ Department of Physics and Astronomy, University of Tennessee, Knoxville, TN 37996, USA.; ^5^ Min H. Kao Department of Electrical Engineering and Computer Science, University of Tennessee, Knoxville, TN 37996, USA.; ^6^ Department of Physics, Massachusetts Institute of Technology, Cambridge, MA 02139, USA.; ^7^ DEVCOM Army Research Laboratory, Adelphi, MD 20783, USA.

## Abstract

Polar metals are challenging to identify spectroscopically because the fingerprints of electric polarization are often obscured by the presence of screening charges. Here, we unravel unambiguous signatures of a distortive polar order buried in the Fermi sea by probing the nonlinear optical response of materials driven by tailored terahertz fields. We apply this strategy to investigate the topological crystalline insulator Pb_1−*x*_Sn*_x_*Te, tracking its soft phonon mode in the time domain and observing the occurrence of inversion symmetry breaking as a function of temperature. By combining measurements across the material’s phase diagram with ab initio calculations, we demonstrate the generality of our approach. These results highlight the potential of terahertz driving fields to reveal polar orders coexisting with itinerant electrons, thus opening additional avenues for material discovery.

## Teaser

Nonlinear terahertz spectroscopy unravels polar orders buried in metallicity.

## INTRODUCTION

Ferroelectrics are solids with a spontaneous electric polarization whose polarity can be switched by an applied electric field (*1*, *2*). Ferroelectricity typically emerges in electrical insulators with localized electrons that are tightly bound to atoms. In metals, the presence of itinerant electrons leads instead to efficient screening of the electrostatic interionic forces, quenching the long-range dipole-dipole interactions necessary for ferroelectric ordering. For this reason, ferroelectricity and metallicity have long been considered seemingly incompatible material properties.

In 1965, Anderson and Blount (*3*) challenged this notion by proposing that a nominally first-order ferroelastic transition in a metal could acquire second-order character through a displacive (ferroelectric-like) component. Since then, substantial progress has been made in the theoretical classification of materials combining polar and metallic properties (*4*–*6*). The term “polar metal” has been coined to describe materials belonging to a polar crystal class and presenting high electrical conductivity. When the emergence of polarity is accompanied by an inversion symmetry–lowering phase transition, the system evolves into a “distortive polar metal.” Conversely, the designation “ferroelectric metal” is reserved for conducting systems demonstrating switchable electric polarization. Notable examples of materials conforming to this classification include the distortive polar metal LiOsO_3_, which exhibits an order-disorder transition (*7*–*9*), and the anisotropic ferroelectric metal WTe_2_, where metallicity and switchable electric polarization are spatially decoupled along orthogonal directions (*10*–*12*). The terminology has been expanded further to encompass materials such as Ca_3_Ru_2_O_7_, which display local-ordering phenomena alike those of distortive polar metals but fail to exhibit an inversion symmetry–lowering phase transition (*13*–*16*). More recently, the discovery of systems combining broken inversion symmetry and nontrivial band topology (*17*–*22*) has spurred interest in the possible coexistence of metallicity and polar orders.

Probing the emergence of polar or ferroelectric orders in metallic materials is a potential route to the identification of exotic functionalities. For instance, these states may show a complex interplay with superconductivity (*23*, *24*) or serve as building blocks for switchable thermoelectric/multiferroic devices (*25*, *26*). However, direct spectroscopic identification of polar orders buried in the Fermi sea has been challenging to date. Experimental methods such as neutron/electron/x-ray scattering (*7*, *27*), piezoresponse force microscopy (*11*), and extreme ultraviolet second harmonic generation (*28*) have provided preliminary insights into the physics of these systems but require large-scale facilities, extended crystal sizes, or sophisticated sample fabrication tools. Therefore, developing contactless, tabletop techniques that enable facile scrutiny of candidate materials remains a primary task in the field.

Here, we reveal unambiguous signatures of a distortive polar metal by combining tabletop terahertz driving fields and optical probes. Our approach relies on resonantly driving a candidate material’s soft mode with a strong terahertz field and detecting its temporal dynamics with optical measurements of second harmonic generation and reflectance. This enables direct observation of zone-center mode softening and changes in collective mode selection rules at the inversion symmetry–breaking transition temperature, unveiling the emergence of a distortive polar order. As this technique is applicable to a broad class of distortive polar and ferroelectric metals ranging from bulk crystals to atomically thin films, it holds a tremendous potential for material discovery.

**Fig. 1.**
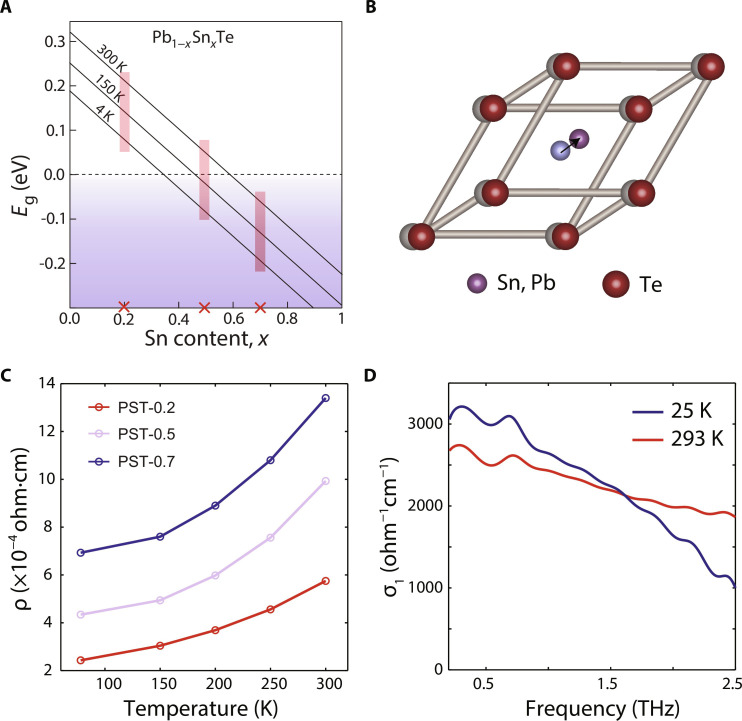
Phase diagram of PST and characterization of metallicity. (**A**) Phase diagram of PST at various temperatures and Sn contents. The red crosses label the doping of the three samples used. Positive gaps refer to the topologically trivial phase, whereas negative (inverted) gaps refer to the TCI phase. Adapted from (*30*). (**B**) Inversion symmetry breaking in PST involves a relative shift of the Sn/Pb and Te atoms along the [111] direction. (**C**) Temperature-dependent electrical characterization of the resistivity of the PST-0.2, PST-0.5, and PST-0.7 samples. The values and trends are consistent with metal-like conduction. (**D**) Real part of the terahertz conductivity for PST-0.5 at two representative temperatures. A broad metallic response is observed with no distinctive phonon signature, in agreement with previous reports (*41*).

We explore this protocol in the Pb_1−*x*_Sn*_x_*Te (PST) class of topological crystalline insulators (TCIs). These materials have a rock-salt unit cell structure at high temperatures (space group *Fm*$\bar{3}$*m*) and undergo a temperature-driven topological phase transition from a trivial insulator to a TCI as the temperature is lowered, with the critical temperature *T*_TCI_ strongly dependent on the doping level *x* (Fig. 1A) (*29*, *30*). The fingerprint of this topological phase transition is the appearance of two-dimensional massless Dirac topological surface states (TSSs), which are protected by mirror symmetry about the crystallographic (110) plane (*29*). When this point-group symmetry is broken spontaneously or by external perturbations, a subset of TSS is gapped out, and the previously massless Dirac fermions acquire a finite mass (*31*–*34*). The presence of a rhombohedral distortion along the [111] direction (Fig. 1B) is the most studied phenomenon behind TSS gapping (*31*). In semiconducting PST alloys, such a polar distortion is caused by the softening of a transverse optical phonon mode (*27*, *35*–*37*). However, still unclear is the evolution of this displacive phase transition in the context of highly metallic PST solid solutions, as the polarization becomes masked by the substantial screening charges introduced by the imperfect stoichiometry and the residual gapless TSS (*38*–*40*).

## RESULTS

In our experiments, we use high-quality (111)-oriented PST films grown on top of GaAs substrates by molecular beam epitaxy (MBE). We focus on three different compositions: *x* = 0.2 (PST-0.2), *x* = 0.5 (PST-0.5), and *x* = 0.7 (PST-0.7). These samples span three different regions in the TCI phase diagram of Fig. 1A: PST-0.2 is topologically trivial at all temperatures explored in our measurements, PST-0.5 lies in the intermediate region where *T*_TCI_ ~ 110 K, and PST-0.7 remains in the TCI state at all temperatures explored (*30*). Because of imperfect stoichiometry, itinerant charge carriers are introduced at all temperatures, making it crucial to systematically analyze the temperature-dependent sample responses to disentangle contributions from various physical processes.

First, we show that these systems exhibit metallic behavior at all temperatures. To accomplish this, we combine electrical transport and time-domain terahertz spectroscopy using a weak terahertz probe. Figure 1C shows the DC longitudinal resistivity for the three compounds in the 77- to 300-K temperature range. The resistivity value on the order of 10^−4^ ohm·cm and the positive correlation between resistivity and temperature (*d*ρ/*dT* > 0) are both indicative of a conducting state. Hall resistivity measurements further allow us to establish carrier densities larger than 10^19^ cm^−3^ and mobilities on the order of 400 to 800 cm^2^/(V·s) (see note S2). Figure 1D displays the real part of the terahertz conductivity (σ_1_) for PST-0.5 at two representative temperatures. It consists of a rather broad Drude feature, in agreement with previous reports using similar samples (*41*). An analysis of the Drude partial spectral weight (see note S5) indicates that the primary source of the metallicity is itinerant carriers from the imperfect stoichiometry. There is no evidence of zone-center transverse optical phonons in the σ_1_ response measured in equilibrium (*36*, *37*), as these modes are likely buried under the Drude conductivity at all temperatures. This raises the question of whether complete phonon softening still occurs in samples that exhibit strong metallic conductivity.

**Fig. 2.**
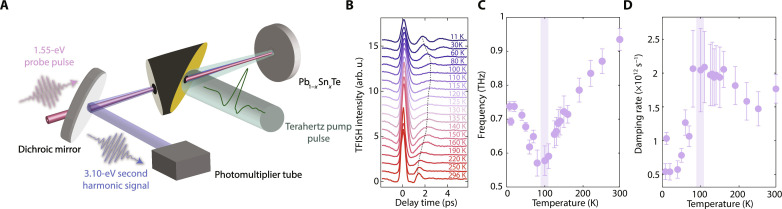
TFISH measurements for PST-0.5. (**A**) Experimental setup for the TFISH measurements. (**B**) Temperature-dependent TFISH time-domain signals for PST-0.5. Phonon oscillations persist after the main terahertz peak response. The oscillation period first increases and then decreases as the temperature is reduced, peaking at ~100 K. This behavior is the hallmark of an inversion symmetry-breaking phase transition. The dashed line connects all the traces in proximity to a crest of the phonon oscillation and is a guide to the eye. The peak terahertz field strength used in the experiment was 560 kV/cm. The traces are vertically offset by a constant for clarity. arb. u., arbitrary units. (**C**) Temperature dependence of the soft mode frequencies. The data are based on fitting the TFISH signal using the equation of motion for a driven damped harmonic oscillator. The low-temperature results also include additional data shown in fig. S8, where the field strength is 630 kV/cm. The frequency of the phonon mode does not vary as a function of terahertz field strength, while the amplitude scales linearly (fig. S10). (**D**) Temperature dependence of the soft mode damping rate.

Therefore, we search for the fingerprint of the soft phonon mode in the time domain using intense terahertz excitation. We generate single-cycle terahertz pulses with peak amplitudes in the range of 100 to 620 kV/cm and focus them on the PST thin films (*42*). Next, we map the terahertz field–induced second harmonic (TFISH) response of a delayed near-infrared probe pulse. A schematic illustration of the setup is presented in Fig. 2A (see note S4 for details). This highly sensitive technique measures the time-dependent hyper-Raman polarizability of the sample upon resonant excitation of a transverse optical phonon mode (*43*), providing access to the dynamics of centrosymmetry-breaking polar lattice modes. Figure 2B shows the TFISH time traces for PST-0.5 at different temperatures and a terahertz field strength of 560 kV/cm. All signals exhibit an initial electronic response that tracks the terahertz pump waveform instantaneously, followed by a damped oscillation that persists after the terahertz field is over. This oscillation is the hallmark of a collective mode coherently evolving in time. Direct inspection of the traces reveals that the mode oscillation is well defined at high (>135 K) and low (<60 K) temperatures but becomes nearly overdamped in the intermediate temperature range (details of the fits are included in note S7). It is also apparent that the oscillation period increases as the temperature approaches 120 K; upon further cooling below this temperature, the oscillation becomes less heavily damped with a faster period.

We extract more quantitative information about the mode dynamics by fitting the time-domain phonon response with a driven damped oscillator equation of motion. This allows us to track the mode frequency (Fig. 2C) and damping rate (Fig. 2D) at different temperatures (for details of the fit and the mode frequency evaluation, see note S7). The mode frequency at 296 K is 0.92 THz and continuously softens toward zero as the temperature approaches the critical temperature *T*_C_ ~ 100 K. The shaded area in the graph indicates the region where the fit becomes less precise as the mode frequency approaches zero. At *T < T*_C_, the mode hardens again. As the driving terahertz field can only access zone-center collective modes, this behavior indicates that the observed oscillation represents the key collective phonon motion associated with an inversion symmetry–lifting phase transition, specifically from a nonpolar to a polar phase. On the basis of the crystallographic orientation and the characteristic mode frequency, we can assign the soft mode to the transverse optical phonon TO_1_. Its almost complete softening at *T*_C_ denotes that the distortive polar phase transition is nearly second order in character. This is confirmed by a fit of the temperature-dependent phonon frequency just below *T*_C_ with Landau’s model, which yields a critical exponent β = 0.19 ± 0.09 (details are given in note S6) (*44*). The damping rate of the mode is also highly suggestive of a distortive polar phase transition: It increases as the temperature decreases from 296 K to *T*_C_, followed by a rapid drop at lower temperatures. This can be explained in terms of enhanced anharmonic coupling between the soft mode and acoustic phonons rather than through electron–soft mode interaction (*27*).

**Fig. 3.**
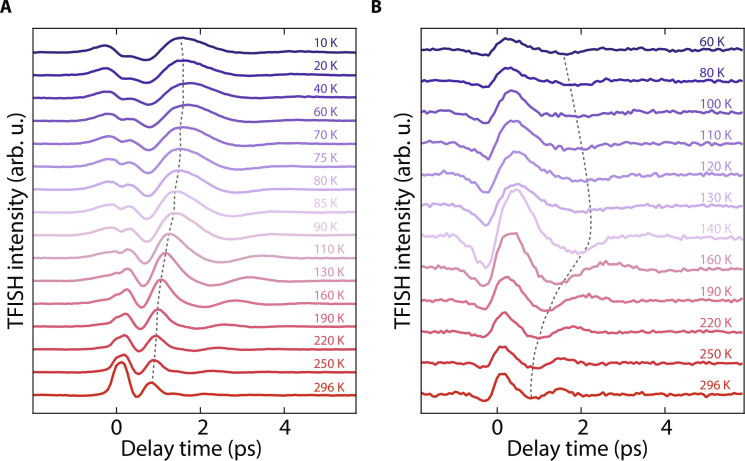
TFISH measurements for PST-0.2 and PST-0.7. Temperature-dependent TFISH time-domain signals for (**A**) PST-0.2 and (**B**) PST-0.7. The dashed lines connect all the traces in proximity of a crest of the phonon oscillation and are guides to the eye. The phonon mode observed in PST-0.2 softens continuously down to the lowest temperatures, while the phonon mode observed in PST-0.7 undergoes softening, followed by a weak sign of hardening at low temperatures. The peak terahertz field strength used was 560 kV/cm. The traces are vertically offset by a constant for clarity.

We extend our measurements to PST-0.2 and PST-0.7. The results are shown in Fig. 3 (A and B), respectively. In PST-0.2, the detected phonon mode softens continuously down to the lowest temperature measured and shows no sign of hardening. This trend is consistent with the fact that PST-0.2 is proximate to the parent compound PbTe, which is an incipient ferroelectric (*45*, *46*). Therefore, the system remains paraelectric at all temperatures and is on the verge of a polar instability. On the other hand, PST-0.7 is close to the parent compound SnTe, which has a polar transition temperature of ~98 K in the bulk form (*35*). Mode softening is observed down to ~120 K, followed by a weak sign of mode hardening at lower temperatures. However, signals at *T* < 60 K are below the sensitivity limit of our apparatus, likely because PST-0.7 is topologically nontrivial at all temperatures and incident terahertz fields are heavily screened by the TSS.

**Fig. 4.**
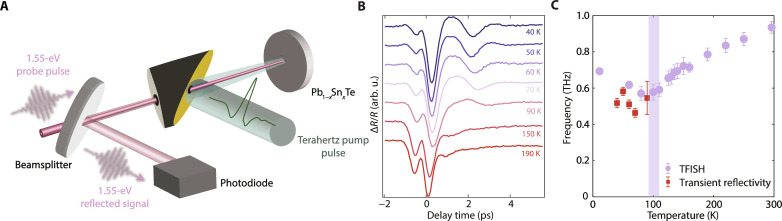
Terahertz-field driven transient reflectivity in PST-0.5. (**A**) Experimental setup for terahertz field–driven transient reflectivity measurements. (**B**) Terahertz field–induced changes in the 1.55-eV reflectivity (Δ*R*/*R*) in PST-0.5. The peak terahertz field strength used in the experiment was 640 kV/cm. The traces are vertically offset by a constant for clarity. (**C**) The temperature-dependent phonon frequencies in PST-0.5 extracted from the terahertz field–driven transient reflectivity and TFISH measurements show a consistent trend.

To further validate the existence of a distortive polar order, we examine the terahertz field–driven dynamics through complementary optical reflectivity measurements, which probe the coherent evolution of Raman-active collective modes (Fig. 4A). At high temperatures, inversion symmetry of PST-0.5 is preserved, and the infrared-active soft mode is silent in transient reflectivity. Upon cooling below *T*_C_, inversion symmetry is broken in the rhombohedral phase, and the zone-center soft phonon mode becomes both infrared and Raman active. This allows for the observation of coherent phonon oscillations that reasonably match those measured by TFISH (Fig. 4, B and C), confirming that transient reflectivity is a sensitive probe of inversion symmetry breaking. Thus, the complementary measurements of second harmonic generation and reflectivity upon terahertz excitation demonstrate that resonant driving of the soft phonon can be used to detect distortive polar states buried in metallicity. We should note that driving the material with pump pulses in the optical range would not lead to the same result. This is because such a photoexcitation scheme cannot initiate coherent dynamics for infrared-active bulk phonon modes in the high-temperature (centrosymmetric) crystal structure (*47*). The use of ultrafast laser pulses at near-infrared/visible photon energies would only lead to the coherent generation and detection of the low-temperature Raman-active phonon response (*48*–*53*), requiring the use of computational calculations to confirm the presence of a distortive polar instability.

## DISCUSSION

Last, we closely examine the action of our terahertz pump pulse in the explored field strength regime. Our goal is to verify that the terahertz field–induced coherent phonon displacements in PST do not trigger a transient or metastable topological phase transition that could complicate the application of our protocol. To this aim, we explore the correlation between the polar distortion along the [111] direction and the topological nature of PST by performing frozen-phonon density functional theory (DFT) calculations on the crystal structure of the SnTe parent compound. Our analysis reveals that lattice displacements of several picometers lead to a rich topological phase diagram, which includes Weyl semimetal, strong topological insulator, and trivial insulator phases (details are provided in note S9). In our experiments, by using the equation of motion of the soft mode under the influence of a terahertz pulse, we estimate an oscillation amplitude on the order of 0.1 pm, a value that is substantially smaller than the excursion required to modify the topological character of our material. This demonstrates that our nonlinear optical spectroscopy method at moderate terahertz field strengths is a feasible tool to investigate the distortive polar instability in the original topological phase of PST. Such a conclusion is further supported by our TFISH data, which exhibits no threshold behavior when the incident terahertz field strength is varied (see note S7).

In summary, we unveiled the emergence of a distortive polar order in PST TCI thin films despite strong free-carrier screening. Specifically, we provided direct evidence of an inversion symmetry–lifting phase transition in PST with high Sn content upon decreasing temperatures. In contrast, low-Sn PST behaved similar to an incipient ferroelectric with no complete phonon softening down to zero temperature. Future implementations of phase-sensitive second harmonic generation in the presence of a static electric field could clarify whether the observed distorted polar metal in PST exhibits a switchable polarization (*54*). The approach described here can be extended to identify inversion symmetry–breaking phase transitions in various types of polar and ferroelectric metals with displacive, order-disorder, or mixed characters (*55*, *56*). In these compounds, polar vector or scalar mode oscillations can be efficiently launched by terahertz excitation and detected with a time-delayed optical probe. We anticipate that our nonlinear optical method will enable the discovery of distortive polar metals with exotic properties such as magnetoelectric couplings and nontrivial topology, paving the way for the development of next-generation devices with simultaneous electrical, magnetic, and optical functionalities.

## MATERIALS AND METHODS

### PST sample synthesis

(111)-oriented PST epitaxial films with a thickness of 325 nm were grown on semi-insulating GaAs (111) substrates using MBE. This orientation was selected for its ability to produce rich topological states at the Γ and *M* points of the Brillouin zone, which are symmetrical with respect to the (110) mirror planes. Furthermore, this orientation enabled facile strain relaxation from dislocation glide along inclined (100) planes. The growth conditions were carefully controlled to ensure a simple (1 × 1) reconstruction of the surface during the entire growth process. Unlike the conventional MBE method that uses PbTe and SnTe compound sources (*57*, *58*), our approach used individual elemental sources (e.g., Pb, Sn, and Te) with >99.9999% purity to precisely control the composition of our materials. Following the growth of PST, an epitaxial BaF_2_ layer was immediately deposited in situ to protect the pristine PST surfaces. This technique allowed for excellent control of the nucleation and alloy composition, producing remarkably smooth surfaces with <1-nm roughness and sharp heteroepitaxial interfaces at both the substrate and BaF_2_ interfaces.

### Linear terahertz transmission spectroscopy

Time-domain terahertz spectroscopy was performed in a transmission geometry. Weak single-cycle terahertz pulses were generated in a photoconductive antenna using 1.55-eV pump pulses from a Ti:sapphire oscillator and transmitted through the PST thin film on a GaAs substrate. Transmitted field signals through the substrate with [*E*_sam_(*t*)] and without the sample [denoted as *E*_sub_(*t*)] were measured in the time domain using another photoconductive antenna and Fourier-transformed to obtain the complex transmission spectra *T*(ω)= *E*_sam_(ω)*/E*_sub_(ω). Additional information regarding the data analysis can be found in notes S4 and S5.

### Second harmonic generation polarimetry

For static second harmonic generation (SHG) measurements, an amplified Yb:KGW laser (Light Conversion, CARBIDE 40 W) was used to generate 270-fs pulses centered around a photon energy of 1.20 eV. The laser source’s repetition rate was 100 kHz. A motorized half-wave plate was inserted in the beam path to rotate the incoming beam’s polarization and collect SHG polarimetry patterns. The sample was positioned within a helium-cooled closed-cycle cryostat (Quantum Design, OptiCool) with a temperature range from 1.6 to 350 K. Nanopositioners (Attocube, ANPx101/LT-linear x-nanopositioner) with subnanometer precision were used to control the sample position. To focus the light onto the sample and collect the optical signal, a 50× objective (Mitutoyo, MY50X-825; numerical aperture, 0.42) was used, giving an optical spot size of ~1 μm. The SHG beam reflected from the sample was directed to an analyzer, which selected either the parallel- or crossed-polarized SHG signal, corresponding to polarization parallel or orthogonal to the incident beam. After filtering out the fundamental light, the SHG signal was detected and read out by a photomultiplier tube (Hamamatsu, H9305-01) coupled to a lock-in amplifier (Zürich Instruments, UHFLI). Additional information regarding the data analysis can be found in note S3.

### TFISH and transient reflectivity spectroscopy

The TFISH and transient reflectivity measurements were performed using a train of 7 mJ, 35 fs pulses produced by a Ti:sapphire amplifier operating at a repetition rate of 1 kHz. The setup used for the TFISH experiments is presented in fig. S5. TFISH was conducted in a reflection geometry using 1.55-eV probe pulses at normal incidence. The second harmonic beam of the reflected probe beam was detected using a photomultiplier tube. In TFISH, the change in the second harmonic intensity of the probe pulses, denoted as $\Delta I_{2\omega }^{\text{TFISH}}$ , was measured by subtracting the static second harmonic background intensity (i.e., second harmonic signals with terahertz pulses blocked) from the detected second harmonic signals (using pump chopping). For the transient reflectivity measurements, the intensity of the 1.55-eV probe beam was first adjusted using a half-wave plate and a polarizer. The reflected probe light from the sample was then redirected by a beamsplitter to a photodiode. The change in the reflectance, denoted as Δ*R*, was measured by subtracting the static reflectance from the detected reflectance (using again pump chopping). The Δ*R* signal was then divided by the static reflectance to yield the Δ*R*/*R* response.

### DFT calculations

DFT calculations were conducted through the Vienna Ab initio Simulation Package (VASP) (*59*, *60*) to obtain the most stable structure, phonon properties, and dipole moment. The projector augmented wave pseudo-potentials have been adopted to describe the valence electron for Sn and Te. The Perdew-Burke-Ernzerhof functional has been used to treat electronic interactions. The energy cutoff of plane waves was set to be 500 eV, and the width of the Gaussian smearing method was chosen to be 0.02 eV. A Γ-centered *k* mesh of 13 × 13 × 13 was used for the Brillouin zone integrations with a resolution of 0.02 2π/Å. To obtain the ground state, the convergence criteria of lattice optimizations were set to 10^−7^ eV and 0.1 meV/Å for total energy and ionic forces, respectively. The density functional perturbation theory implemented in VASP was performed to obtain the force constants with a 4 × 4 × 4 supercell for phonon dispersion by Phonopy code (*61*). Additional DFT calculations were performed with the full-potential local-orbital program. The symmetry-adapted Wannier tight-binding models were constructed by projecting the Bloch wave function to localized Wannier functions. To identify various topological semimetals phases, the entire Brillouin zone was scanned searching for degenerate points, and the Chern number was calculated over the sphere enclosing them. For topological insulators and TCI phases without inversion symmetry, Wilson loop calculations were performed to obtain the *Z*_2_ index and the Berry curvature integral (over wave function manifold indexed by the mirror eigenvalue to obtain the mirror Chern number). Additional information regarding the first-principles calculations can be found in note S9.

## Supplementary Materials

### This PDF file includes:

Supplementary Text Figs. S1 to S18 Table S1 References
