# Supplementary material for: Revealing a distortive polar order buried in the Fermi sea
Source: Sci Adv. 2024 Jul 12;10(28):eadn0929. doi: 10.1126/sciadv.adn0929 (PMC11244435; doi:10.1126/sciadv.adn0929)
Supplement: Supplementary file 1 — Supplementary Text Figs. S1 to S18 Table S1 References [file sciadv.adn0929_sm.pdf]

Science Advances
AAAS

Supplementary Materials for

Revealing a distortive polar order buried in the Fermi sea

Jiaojian Shi *et al.*

Corresponding author: Edoardo Baldini, edoardo.baldini@austin.utexas.edu

Sci. Adv. **10**, eadn0929 (2024)
DOI: 10.1126/sciadv.adn0929

This PDF file includes:

Supplementary Text
Figs. S1 to S18
Table S1
References

Supplementary Text

Supplementary Note 1: Sample growth and characterization

(111)-oriented $Pb_{1-x}Sn_xTe$ epitaxial films were grown on semi-insulating GaAs (111) substrates using molecular beam epitaxy (MBE). This orientation was selected for its ability to produce rich topological states at the Γ and M points of the Brillouin zone, which are symmetrical with respect to the (110) mirror planes. Furthermore, this orientation enabled facile strain relaxation from dislocation glide along inclined (100) planes. The growth conditions were carefully controlled to ensure a simple (1×1) reconstruction of the surface during the entire growth process, as evidenced by the Reflection High Energy Electron Diffraction (RHEED) pattern taken along the [110] azimuth (Fig. S1).

Unlike the conventional MBE method that uses PbTe and SnTe compound sources (*57, 58*), our approach employed individual elemental sources (*e.g.*, Pb, Sn and Te) with > 99.9999% purity to precisely control the composition of our materials. Following the growth of $Pb_{1-x}Sn_xTe$, an epitaxial BaF_2 layer was immediately deposited *in situ* to protect the pristine $Pb_{1-x}Sn_xTe$ surfaces. This technique allowed for excellent control of the nucleation and alloy composition, producing remarkably smooth surfaces with < 1 nm roughness and sharp heteroepitaxial interfaces at both the substrate and BaF_2 interfaces. This is demonstrated in Fig. S2, which shows transmission electron microscopy (TEM) images of the two interfaces.

MBE layers were grown with varying compositions, including $Pb_{0.8}Sn_{0.2}Te$ (PST-0.2), $Pb_{0.5}Sn_{0.5}Te$ (PST-0.5), and $Pb_{0.3}Sn_{0.7}Te$ (PST-0.7), to their described thicknesses (325 nm) under Te-rich conditions. The transition temperatures to the topological crystalline insulator and distortive polar phases (T_{TCI} and T_C) for the three compounds are summarized in Table S1.

Supplementary Note 2: Electrical characterization

To confirm that the PST samples that were explored in this work had metal-like transport character, we performed temperature-dependent magnetoresistance measurements from 77 K to 300 K using samples patterned in the simple van der Pauw geometry under a 2 T magnetic field. As shown in Fig. 1C of the main text, the samples exhibited a resistivity on the order of $2\text{-}13\times10^{-4}$ ohm cm, with a positive slope that increased with increasing temperature. These aspects were characteristic of metal-like conduction. While all resistivity values tended to increase by roughly a factor of 2 from 77 K to 300 K, the carrier densities remained somewhat invariant with temperature (Fig. S3A). At 300 K, the mobility values were all quite large and fell in the range of 500 $cm^2/(V\cdot s)$ (Fig. S3B). The decreasing trend of mobility with increasing temperature was also consistent with metal-like conduction (*p*-type).

Supplementary Note 3: Polar phase transition with static second harmonic generation

We used static second harmonic generation (SHG) to track the emergence of the polar distortive phase. The resulting temperature-dependent SHG signals for a pair of (111)-oriented $Pb_{1-x}Sn_xTe$ samples (PST 0.5 and PST 0.7) are shown in Fig. S4A. Across the transition temperature range of 90-100 K, both samples exhibited substantial increases in the static SHG signal, suggesting a

possible inversion symmetry breaking in the material. We then collected a series of temperature dependent SHG polarimetry measurements on the PST-0.7 sample. The results are presented in Fig. S4B. The anisotropic response reveals a reduction in symmetry across the transition temperature, consistent with the loss of the C_3 axis of rotation along the [111] crystal direction and thus cubic symmetry.

To further elucidate the origins of the temperature dependent SHG polar patterns, we apply a simple model for the anisotropic SHG signal, writing the nonlinear polarization as

$$P_i^{2\omega}(T) = \chi_{ijk}^{ED}(T)E_j^{\omega}E_k^{\omega} + \chi_{ijkl}^{EQ}E_j^{\omega}\nabla_k E_l^{\omega},$$

where χ_{ijk}^{ED} represents an electric-dipole SHG response and χ_{ijkl}^{EQ} represents an electric quadrupole SHG response. The former is only present below the polar phase transition temperature, scaling with the stabilization of the electric polarization. The latter should be present at all temperatures and relatively temperature insensitive. Starting in the high-temperature cubic phase (point group *m-3m)*, the electric quadrupole tensor χ_{ijkl}^{EQ} has 4 independent elements:

$$\chi_{xxxx}^{EQ} = \chi_{yyyy}^{EQ} = \chi_{zzzz}^{EQ},$$
$$\chi_{xxyy}^{EQ} = \chi_{xxzz}^{EQ} = \chi_{yyxx}^{EQ} = \chi_{yyzz}^{EQ} = \chi_{zzxx}^{EQ} = \chi_{zzyy}^{EQ},$$
$$\chi_{xyxy}^{EQ} = \chi_{xzxz}^{EQ} = \chi_{yxyx}^{EQ} = \chi_{yzyz}^{EQ} = \chi_{zxzx}^{EQ} = \chi_{zyzy}^{EQ},$$
$$\chi_{xyyx}^{EQ} = \chi_{xzzx}^{EQ} = \chi_{yxxy}^{EQ} = \chi_{yzzy}^{EQ} = \chi_{zxxz}^{EQ} = \chi_{zyyz}^{EQ}.$$

We consider a back-reflection geometry with incident $\mathbf{k}^{\omega}$ light and outgoing $\mathbf{k}^{2\omega}$ waves propagating anti-parallel and parallel to the out-of-plane [111] axis, respectively. The anisotropic SHG response in the parallel- and cross-polarized detection channels can be written as:

$$P_{\parallel}^{2\omega,EQ}(\phi) = \left(\chi_{xxxx}^{EQ} - \chi_{xxyy}^{EQ} - \chi_{xyyx}^{EQ} - \chi_{xyxy}^{EQ}\right)\cos 3\phi,$$
$$P_{\perp}^{2\omega,EQ}(\phi) = \left(\chi_{xxxx}^{EQ} - \chi_{xxyy}^{EQ} - \chi_{xyyx}^{EQ} - \chi_{xyxy}^{EQ}\right)\sin 3\phi,$$

where the azimuthal angle ϕ is measured relative to the in-plane [-211] axis. When converted to SHG intensities, the resulting six-fold symmetric polar patterns closely approximate the SHG signal found at high temperatures, signaling the lack of inversion symmetry breaking. Upon cooling below the transition temperature, a distinct C_2 axis emerges in the polarimetry plots, signifying the emergence of an electric polarization in the material. Assuming this polarization appears along the [100] axis, the new tetragonal crystal structure (point group *4mm*) allows three independent electric-dipole tensor elements for a degenerate SHG process:

$$\chi_{xxx}^{ED}, \chi_{xyy}^{ED} = \chi_{xzz}^{ED}, \chi_{yyx}^{ED} = \chi_{yxy}^{ED} = \chi_{zzx}^{ED} = \chi_{zxz}^{ED}.$$

Writing the corresponding nonlinear responses in the experimental detection geometry we have

$$P_{\parallel}^{2\omega,ED}(\phi) = \frac{3}{2}\left(2\chi_{yyx}^{ED} + \chi_{xyy}^{ED}\right)\cos\phi + \left(\chi_{xxx}^{ED} - 2\chi_{yyx}^{ED} - \chi_{xyy}^{ED}\right)\cos^3\phi,$$
$$P_{\perp}^{2\omega,ED}(\phi) = \left(\chi_{xxx}^{ED} + \frac{1}{2}\chi_{xyy}^{ED} - 2\chi_{yyx}^{ED}\right)\sin\phi - (\chi_{xxx}^{ED} - 2\chi_{yyx}^{ED} - \chi_{xyy}^{ED})\sin^3\phi.$$

Combining this with the above quadrupolar response, we can write the overall temperature-dependent anisotropic SHG signal as

$$I_{\parallel}^{2\omega}(\phi,T) = \left|P_{\parallel}^{2\omega,ED}(\phi,T) + P_{\parallel}^{2\omega,EQ}(\phi)\right|^2 + C$$
$$I_{\perp}^{2\omega}(\phi,T) = \left|P_{\perp}^{2\omega,ED}(\phi,T) + P_{\perp}^{2\omega,EQ}(\phi)\right|^2 + C.$$

The additional parameter C represents an isotropic incoherent background signal. Utilizing this model, we fit the observed polar SHG response and show the fits along with the experimental data in Fig. S4B. The retrieved nonlinear optical susceptibilities are displayed in Fig. S4C. The results exhibit close agreement with the data, with the electric-dipole response prominently attributable to χ_{xxx}^{ED} and χ_{xyy}^{ED}. Thus, the static SHG polarimetry signal is consistent with the observation of an induced electric-dipole SHG response emerging from a material polar distortion.

Supplementary Note 4: Linear THz transmission spectroscopy and THz-field induced second harmonic generation

Time-domain terahertz (THz) spectroscopy was performed in a transmission geometry. Weak single-cycle THz pulses were generated in a photoconductive antenna using 1.55 eV pump pulses from a Ti:Sapphire oscillator and transmitted through the PST thin film on a GaAs substrate. Transmitted field signals through the substrate with [denoted as $E_{sam}(t)$] and without the sample [denoted as $E_{sub}(t)$] were measured in the time domain using another photoconductive antenna and Fourier transformed to obtain the complex transmission spectra $T(\omega) = E_{sam}(\omega)/E_{sub}(\omega)$. For conducting thin films, the complex THz conductivity $\sigma(\omega)$ can be calculated from the transmittance $T(\omega)$ using the Tinkham formula (*59*) as $T(\omega) = (1+n_s)/(1+n_s+\sigma(\omega)Z_0 d_f)$, where n_s is the refractive index of the substrate, Z_0 is the impedance of free space and d_f is the film thickness. The complex THz conductance (*60*) can then be derived from the conductivity as $G(\omega) = \sigma(\omega)d_f$ and fitted with a Drude model.

The experimental setup for THz-field induced second harmonic generation (TFISH) is presented in Fig. S5. The measurements were conducted in a reflection geometry using 1.55 eV probe pulses at normal incidence. The second harmonic (SH) beam of the reflected probe pulses was detected using a photomultiplier tube. In TFISH, the change in the SH intensity of the optical probe pulses, denoted as $\Delta I_{2\omega}^{TFISH}$, was measured by subtracting the static SH background intensity (*i.e.*, SH signals with THz pulses blocked) from the detected SH signals (using pump chopping).

In centrosymmetric materials, TFISH is typically a homodyne measurement because polar displacements along either direction break inversion symmetry and produce finite SH intensity (relative to the otherwise zero SH intensity level). As a result, the detected TFISH signals appear

at twice the natural frequency of the polar process. However, when a finite second-order nonlinearity, or $\chi^{(2)}$, already exists in the sample (*e.g.*, in non-centrosymmetric systems or from surface SH generation), the polar displacements along the two polarities change the total SH intensity in opposite ways. For example, they increase or decrease the total SH intensity relative to its original nonzero level depending on the phase of the THz-induced SH field relative to the background SH field. Consequently, the TFISH signals can appear at their fundamental frequencies. A heuristic equation can be formulated to explain this concept (*61*):

$$\Delta I_{2\omega}^{TFISH} \propto \left(\chi^{(2)} + \chi^{(3)} E_{THz}\right)^2 I_\omega^2 - \left(\chi^{(2)} I_\omega\right)^2 \propto \left[2\chi^{(2)}\chi^{(3)} E_{THz} + \left(\chi^{(3)} E_{THz}\right)^2\right] I_\omega^2, \quad \text{(S1)}$$

where I_ω denotes the intensity of the 1.55 eV probe pulse, and $\chi^{(2)}$ and $\chi^{(3)}$ represent the second- and third-order nonlinear optical susceptibility coefficients, respectively. As shown in Eq. 1, in the presence of the THz field, a negative TFISH signal indicates the existence of a nonzero $\chi^{(2)}$, since signals that arise purely from the $\chi^{(3)}$ contribution must be positive. In this case, TFISH signals appear at the fundamental frequencies of the underlying processes, as discussed above. In our measurements, we clearly observe negative TFISH signals during the presence of the THz field, indicating a finite $\chi^{(2)}$ and thus heterodyne TFISH measurements. It is worth noting that the above discussion about homodyne/heterodyne measurements only affects the quantitative extraction of the soft mode parameters during the curve fitting process and does not change the main conclusion of our paper (*i.e.*, the observation of softening and hardening of the phonon mode).

Supplementary Note 5: Analysis of the THz conductivity in PST-0.5

We analyzed the THz conductivity in PST-0.5 to deduce its main origin. We obtained the plasma frequency ω_p by fitting the real and imaginary parts of $\sigma(\omega)$ at various temperatures to the Drude model. Figure S6 shows the extracted plasma frequency, which is in good agreement with previously reported values for similar compounds *(62)*. To compare the contributions of the topological surface states (TSS) and itinerant electrons due to imperfect stoichiometry, we estimated the partial spectral weight $SW = \int_0^\Omega \sigma_1(\omega) d\omega$, where $\Omega/2\pi$ = 2.6 THz is the experimental cutoff frequency, as shown in Fig. S7. We used the conductivity data from the topological insulator Bi_2Se_3 to estimate the TSS contributions qualitatively *(63)*. The results show that the TSS contribution is several orders of magnitude smaller than the spectral weight calculated from the experiment, indicating that itinerant carriers introduced by imperfect stoichiometry are the dominant origin of the THz conductivity.

Supplementary Note 6: Additional data from TFISH measurements

We performed temperature-dependent TFISH measurements of a (111)-oriented PST-0.5 epitaxial film on a GaAs substrate. The results are shown in Fig. S8. Phonon mode softening and hardening are observed, confirming the existence of a distortive polar phase transition in the material. The soft mode frequencies and damping rates shown in the main text’s Fig. 2 were extracted from the time-domain TFISH data by numerical fitting as described in Supplementary Note 7. The temperature-dependent mode frequency can be described by Landau's theory through the form

$$\omega_0 = \alpha|T - T_c|^{\beta} \tag{S3}$$

where ω_0 is the mode angular frequency, T_C is the transition temperature, T is experimental temperature, and amplitude α and exponent β are fitting parameters. The fit just below T_C yields $\beta = 0.19\pm0.09$, while the fit above T_C yields $\beta = 0.11\pm0.04$. See Fig. S9 for the details. The phonon softening around 100 K is a fingerprint of a distortive polar phase transition. The parent compound SnTe is a well-known distortive polar material, and signatures of polarity have been found for finite Pb content (x = 0.25 and x = 0.5) (*64*). For $Pb_{0.5}Sn_{0.5}Te$ single crystals, the estimated distortive polar transition temperature was 62-70 K. We remark that variations in carrier density and sample type (*i.e.*, bulk crystal vs. thin film) can introduce changes in the transition temperature. For example, the samples used in Ref. (*64*) are single crystals that are slightly doped with In to pin the chemical potential, while ours are thin films that do not contain any In.

Supplementary Note 7: Numerical fitting of the TFISH time traces

To fit the full traces of THz-induced SHG changes, we used the following three-component expression that includes the non-resonant electronic response as well as the phononic response to the THz field:

$$\Delta I_{2\omega}^{TFISH} \propto \left[\chi_{el} + \chi_{ph} + \chi_{st}\right]^2 I_\omega^2 - \chi_{st}^2 I_\omega^2 \tag{S4}$$

In this expression, χ_{st} is the static contribution of SHG susceptibility at equilibrium, χ_{el} is the instantaneous electronic contribution near time zero imparted by THz electric field $E(t)$ (the experimentally measured THz profile is shown in Fig. S12), and χ_{ph} is the phononic contribution which is described by a driven damped harmonic oscillator equation of motion for the coherent phonon displacement $Q(t)$ with the driving force proportional to $E(t)$ (see Supplementary Note 10). The SHG signal intensity initially decreases from its value prior to the arrival of the excitation pulse, indicating that a static SHG signal was already present. This is described by χ_{st} which takes on a time-independent positive value. The initial decrease of SHG signal followed by a strong increase and subsequent decrease indicates that the initial THz-induced contribution to signal described by $\chi_{el} \propto E(t)$ followed the THz field profile in sign (negative, strongly positive, negative) and suggests that the field-driven phonon contribution to signal $\chi_{ph} \propto Q(t)$ behaves the same way, *i.e.*, the driven phonon response also results in negative, then positive, then negative contributions to the SHG signal intensity. The fits to the data in Fig. S10 and Fig. S11 are shown in Fig. S14 and Fig. S15, respectively. We first fitted the TFISH trace at the highest THz field strength of 620 kV/cm taken at 60 K. This choice was based on the appearance of a kink at approximately 0.7 ps which allowed us to determine the value of the static contribution to the SHG signal. The kink arises because the negative contributions to the SHG signal given by $\chi_{el} + \chi_{ph}$ (as both $E(t)$ and $Q(t)$ become negative after t = 0) barely overtake the static contribution χ_{st}, so the sum $\chi_{el} + \chi_{ph} + \chi_{st}$ very briefly becomes negative. The SHG signal intensity is proportional to $\left[\chi_{el} + \chi_{ph} + \chi_{st}\right]^2$, so as the sum passes through zero twice with a brief negative excursion, the

SHG signal reaches zero twice with a small positive signal level in between the minima. That is what generates the kink-like appearance the data. It is convenient for us because it allows determinaton of the static contribution to the signal with reasonable accuracy, and this contribution remains the same throughout the field-dependent data. The kink only appears at the highest THz field levels. At lower levels the negative-going sum $\chi_{el} + \chi_{ph}$ never overtakes the static contribution χ_{st}, so kink does not appear. To fit selected features of the data such as the kink and the phonon oscillations, we manually adjusted the parameters to yield the best fit to the signal at the highest field strength. The fit is shown in Fig. S14A. We display the numerically calculated driven phonon response along with the electronic response in Fig. S14F. For fits to the data at the lowest field strengths, we assumed that the phonon frequency and damping rate were independent of the strength of the THz driving field, and that the phonon and electronic amplitudes scaled linearly with the measured THz field strength. The resulting fits are shown in Figs. S14B-E.

We subsequently applied the same fitting procedure to the data taken at 295 K (Fig. S11). First, we performed a fit to the high-field trace, obtaining reasonable starting parameters for subsequent manual refinement. Following this, the electronic and phonon components were scaled linearly relative to the THz field to extend the fit to other traces, while the static component remained fixed. The results are shown in Fig. S15, revealing a reasonably good fit for the high-field traces. However, we observed a notable deviation at lower fields, particularly in the vicinity of the time-zero response. This complexity in the response is likely attributed to incipient behavior associated with Dirac electrons, which exhibit nonlinear responses to the THz field (*66*). It is probable that both Dirac and conventional electronic responses contribute to the near-time-zero signal in the non-stoichiometric crystal, with normal linear responses predominating at high fields due to saturation of the former. We note that no threshold-like behavior was observed in the phonon response across varying field strengths, indicating the absence of evidence for a phase transition induced by coherent phonon displacements.

We applied the same general fitting procedure to the temperature-dependent TFISH data of main text's Fig. 2. The results are shown in Fig. S13. Since the phonon parameters and the contribution amplitudes did not remain constant at different temperatures, all the parameters were allowed to vary for these fits.

Finally, we note that such fitting procedure is very general and can reproduce the dynamics of any materials with a phonon mode that lies at THz frequencies. It can also account for a special case of soft-mode frequency doubling when there is no static SHG in the sample. The question whether a coherent collective mode modulates the TFISH response at its natural angular frequency (ω) or at twice the frequency (2ω) depends on the magnitude of the background (static) SHG signal. For example, in Ref. *(67)*, the TFISH response was measured in a transmission geometry through a bulk sample of $SrTiO_3$. Above the transition temperature (T_C), $SrTiO_3$ was centrosymmetric, and there were no important contributions to the static SHG signal (neither from bulk electric-dipole or from surface electric-dipole). As such, the phonon-induced oscillation was the only source of SHG in the TFISH response. Since phonon excursions in either direction had the same effect, the SHG oscillations appeared at 2ω. In the current article, we measured the TFISH response in a reflection geometry on a thin film of $Pb_{1-x}Sn_xTe$. Above T_C, the system was centrosymmetric, like

$SrTiO_3$. Consequently, the background SHG signal had no bulk electric-dipole contribution. However, unlike $SrTiO_3$, $Pb_{1-x}Sn_xTe$ had a significant SHG signal above T_C (see Supplementary Note 3). Therefore, the phonon-induced SHG was heterodyned by the static SHG, and the phonon oscillations caused the measured SHG intensity to alternatively increase and decrease about the static SHG intensity at the natural phonon frequency. The presence of a static SHG signal was appreciated from the behavior of the TFISH response at the highest THz field strengths (*i.e.*, 592 kV/cm and 620 kV/cm in Fig. S10). In those traces, the phonon-induced SHG amplitude became quite strong and overcame that of the static SHG. As a result, the SHG intensity decreased to zero, showed a small "extra peak", decreased almost to zero again, and then continued with oscillations at the phonon frequency ω (because damping reduces the phonon-induced SHG field amplitude to a level below that of the static SHG). The small extra peak at t = 1 ps was the frequency-doubling of the phonon oscillations. This feature allowed us to calibrate the static SHG field amplitude relative to the phonon-induced SHG and the non-resonant electronic response at t = 0 ps. Since the static SHG amplitude did not change as a function of THz field strength, we were able to fit the data at all field strengths using the static amplitude determined from the data recorded with the strongest THz field.

Supplementary Note 8: Analysis transient reflectivity signal

In the context of transient reflectivity measurements, phonon oscillations were exclusively observable at temperatures below the critical temperature, T_C, within the non-centrosymmetric rhombohedral phase. We employed numerical results derived from a driven harmonic oscillator model in the presence of a THz field to fit the transient reflectivity data presented in Fig. 4 of the main text. Only the transient reflectivity signal following time zero was considered, as there were challenges in fitting the data over the entire time span. Figure S16 illustrates the outcomes of the fitting process.

Supplementary Note 9: First-principles calculations

Emergence of a macroscopic polarization: We studied the emergence of a finite macroscopic polarization driven by displacements along the soft phonon mode. SnTe crystalizes in the centrosymmetric cubic rock-salt structure, which belongs to the $Fm\bar{3}m$ space group. The phonon dispersion along high-symmetry lines for SnTe is shown in Fig. S17A. Due to mirror symmetries, the three optical modes are degenerate at the Γ point. The dipole moment for this structure vanishes due to the presence of inversion symmetry. We considered the distortion of the transverse optical (TO) mode, which involves vibrations along the y-direction for both Sn and Te atoms. We calculated the polarization emerging in the system upon displacements along the phonon mode. To this aim, we set the chemical potential inside the material's gap. We found that the TO phonon distortion breaks inversion symmetry, leading to a non-zero dipole moment with value (0.0, -0.713 ,-0.818)eÅ. We also evaluated the phonon dispersion and electric dipole moment for SnTe with a static distortion along the [111] direction. The phonon dispersion, shown in Fig. S17B, reveals that two TO modes along the X-Γ direction split due to the breaking of the mirror symmetry (Mx). In the presence of this distortion along [111], the calculated electric dipole moment becomes (0.885,1.857,-1.366)eÅ.

Phonon-mediated topological phase transitions: We also investigated how the displacements along the polar [111] direction can lead to a rich phase diagram with distinct topologically non-trivial phases. To this aim, we performed DFT calculations of SnTe with the Full-Potential Local-Orbital program (FPLO) (*68*). We then constructed the symmetry-adapted Wannier tight-binding models by projecting the Bloch wavefunction to localized Wannier functions. To identify the presence of topological semimetal phases, we scanned the entire Brillouin zone searching for degenerate points and calculated the Chern number over the sphere enclosing them (*69*). For topological insulators and topological crystalline insulators without inversion symmetry, we performed a Wilson loop calculation to obtain the $\mathbb{Z}_2$ index and Berry curvature integral over the wavefunction manifold indexed by the mirror eigenvalue to obtain the mirror Chern number. From these calculations, we found that SnTe undergoes a rich series of topological phase transitions under polar distortion along the [111] direction (shown in Fig. S18). The temperature dependent distortion ranges from 4.22 pm at zero temperature to 0 pm at the polar transition temperature (*70*). From the centrosymmetric phase, which gives zero SHG signal, to 1.73-pm polar distortion, SnTe remains a TCI with band inversions at both L and Z points of the Brillouin zone. Between 1.73-pm and 1.79-pm polar distortion, the gap at Z closed and Weyl points emerge around Z. When the distortion is between 1.79 pm and 2.75 pm, the gap at Z opens again, and SnTe becomes a strong topological insulator with single Dirac points at the projected (111) surface, verified from the Wilson-loop calculation. When the distortion increases between 2.75 pm and 2.88 pm, the gap at the K points also closes, and SnTe becomes a Weyl semimetal with 12 pairs of Weyl points around L points. As the distortion exceeds 2.88 pm, the gap at the L points opens again, and SnTe becomes a trivial polar insulator without band inversions at Z and L points.

Supplementary Note 10: Estimation of THz-driven soft mode displacement

We estimated the THz-driven soft mode displacement using the driven damped harmonic oscillator model given by the following equation:

$$\ddot{Q} + 2\gamma\dot{Q} + \omega_0^2 Q = Z^* E(t)/\epsilon \qquad \text{(S5)}$$

where Q is the time-dependent atomic displacement, Z^* is the effective charge dipole of the phonon mode, γ is the damping rate, ω_0 is the soft mode angular frequency, ϵ the screening factor, and $E(t) = E_0 \sin(\omega_{THz} t)\, e^{-t^2/T^2}$ is the excitation THz pulse profile. When the driving frequency is resonant with the soft mode frequency $\omega_0/2\pi$, the amplitude A in the multicycle limit is given by $A = \frac{Z^* E_0}{2m\epsilon\gamma\omega_0}$. Based on our experimental observations, we chose $\omega_0/2\pi$ to be about 0.3 - 1 THz and γ to be around 0.15 - 0.4 THz for the PST-0.5 sample at 11 - 296 K. After evaluating the screening factor ϵ through time-domain THz spectroscopy *(41, 71, 72)*, we obtained a final amplitude on the order of 0.1 pm, *i.e.*, ~ 0.01% of the unit cell. This displacement is much smaller than the ones considered in our DFT calculations and indicates that TFISH can characterize the emergence of a distortive polar order without inducing a topological phase transition.

Supplementary Note 11: Additional discussion on the distortive polar phase transition

In our work, we used TFISH and transient reflectivity to identify the symmetry of the mode with respect to spatial inversion. The identification of a soft mode's symmetry is key for clarifying the distortive polar nature of an underlying phase transition. Specifically, since the mode is directly excited by the THz pump pulse in the perturbative regime, it often lies at the Brillouin zone center. Above the phase transition temperature, the mode is only observed in the TFISH channel, indicating its infrared activity. Conversely, below the transition temperature, the mode manifests both in the TFISH and in the transient reflectivity channels, signifying its dual Raman and infrared activity. These findings allow us to conclude that the underlying phase transition must lift inversion symmetry, thus being polar in nature. To rule out other possible origins for the observed phase transition, we first consider charge-density wave (CDW) instabilities. In that case, phonon softening above the transition temperature would occur at finite momentum (*73*), causing the mode to be absent in the TFISH channel. Below T_{CDW}, the amplitude mode would be Raman active, while the phase mode would be infrared active (*74*). Similar considerations apply to the soft phonon of an antiferroelectric phase transition, which softens at the Brillouin zone corner above the transition temperature and would not manifest in our probes (*75*). The only other plausible phase transition that aligns with the behavior we observe is an excitonic insulator transition, where the soft mode is electronic in nature. However, the metallic nature of $Pb_{1-x}Sn_xTe$ rules out the possibility of hosting an excitonic insulator state. Furthermore, the collective mode that we launch with our THz pulse has already been characterized in the SnTe parent compound (*27*).

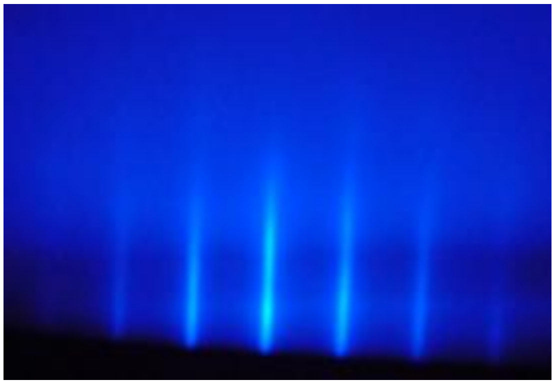

Fig. S1. RHEED characterization of $Pb_{1-x}Sn_xTe$. RHEED image showing (1×1) surface reconstruction during the MBE growth of (111) $Pb_{1-x}Sn_xTe$.

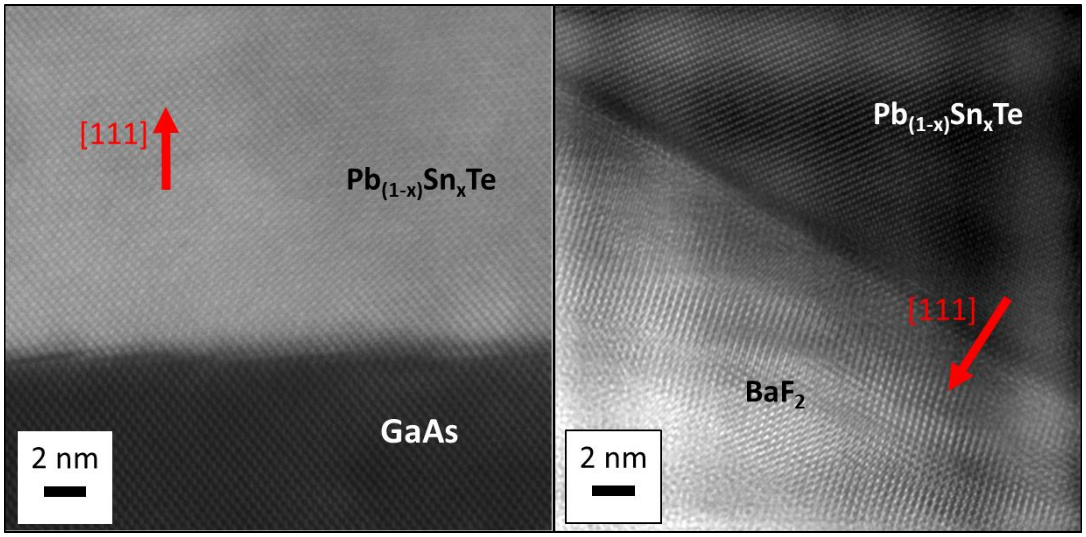

Fig. S2. TEM characterization of $Pb_{1-x}Sn_xTe$. TEM images of the $Pb_{1-x}Sn_xTe$/GaAs and BaF_2/$Pb_{1-x}Sn_xTe$ interfaces.

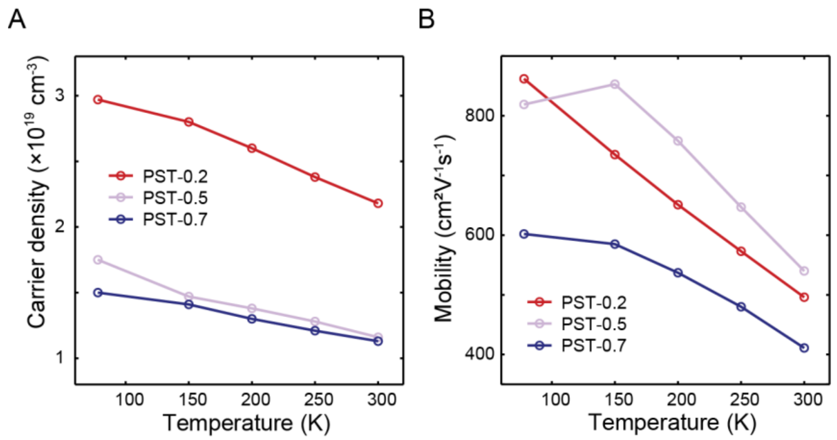

Fig. S3. Electrical characterization of $Pb_{1-x}Sn_xTe$. Temperature-dependent electrical characterization of the (**A**) carrier density and (**B**) mobility of the PST-0.2, PST-0.5, and PST-0.7 samples. The values and trends are consistent with *p*-type metal-like conduction.

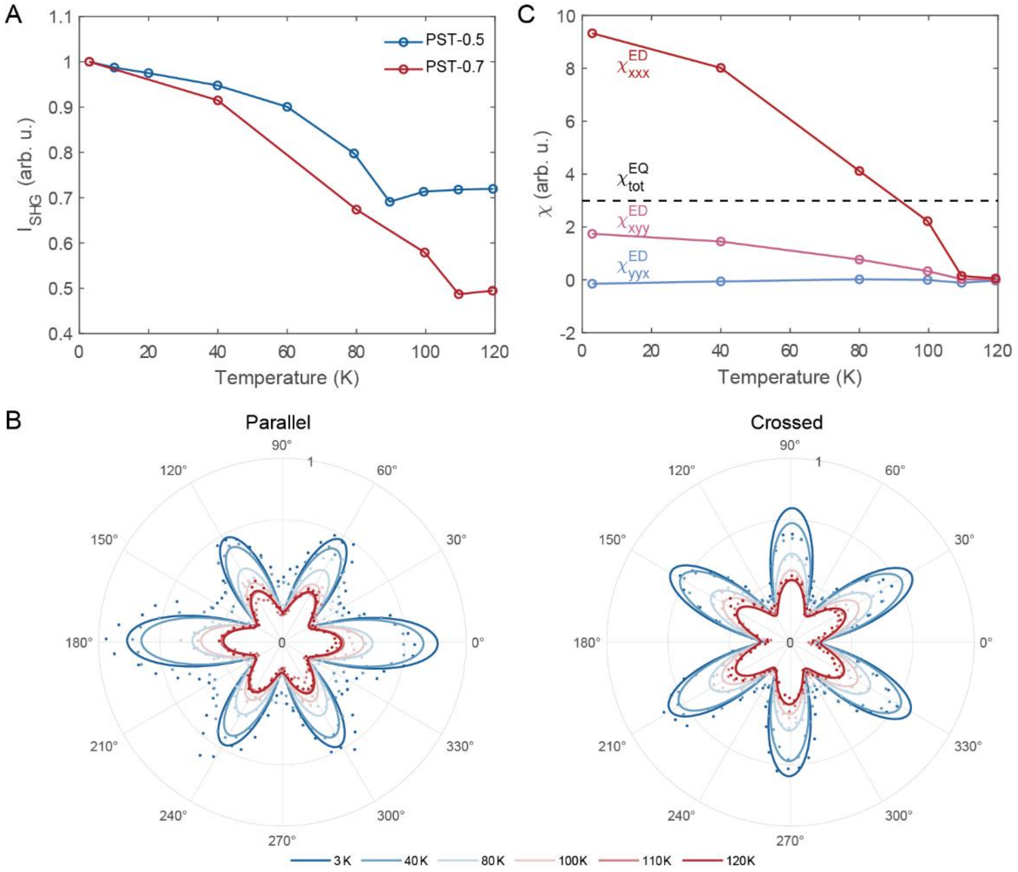

Fig. S4. SHG polarimetry of $Pb_{1-x}Sn_xTe$. (**A**) Temperature-dependent static SHG signal. Experimental temperature-dependent static SHG signals collected from two [111]-oriented samples: PST-0.5 (*blue*) and PST-0.7 (*red*). Values are averaged over a large number of incident probe polarizations $\boldsymbol{\phi}$. (**B**) Temperature-dependent SHG polarimetry. Anisotropic SHG polar patterns (*dots*) collected from PST-0.7 in both parallel- (*left*) and cross- (*right*) polarized detection channels. Also shown are fits (*solid lines*) of the data to the SHG model in Supplementary Note 3. (**C**) SHG polarimetry fitting parameters. Fitted temperature-dependent second-order nonlinear optical susceptibilities for the polarimetry patterns shown in panel (B) using the SHG model presented in Supplementary Note 3. Note that $\boldsymbol{\chi_{tot}^{EQ} = \chi_{xxxx}^{EQ} - \chi_{xxyy}^{EQ} - \chi_{xyyx}^{EQ} - \chi_{xyxy}^{EQ}}$.

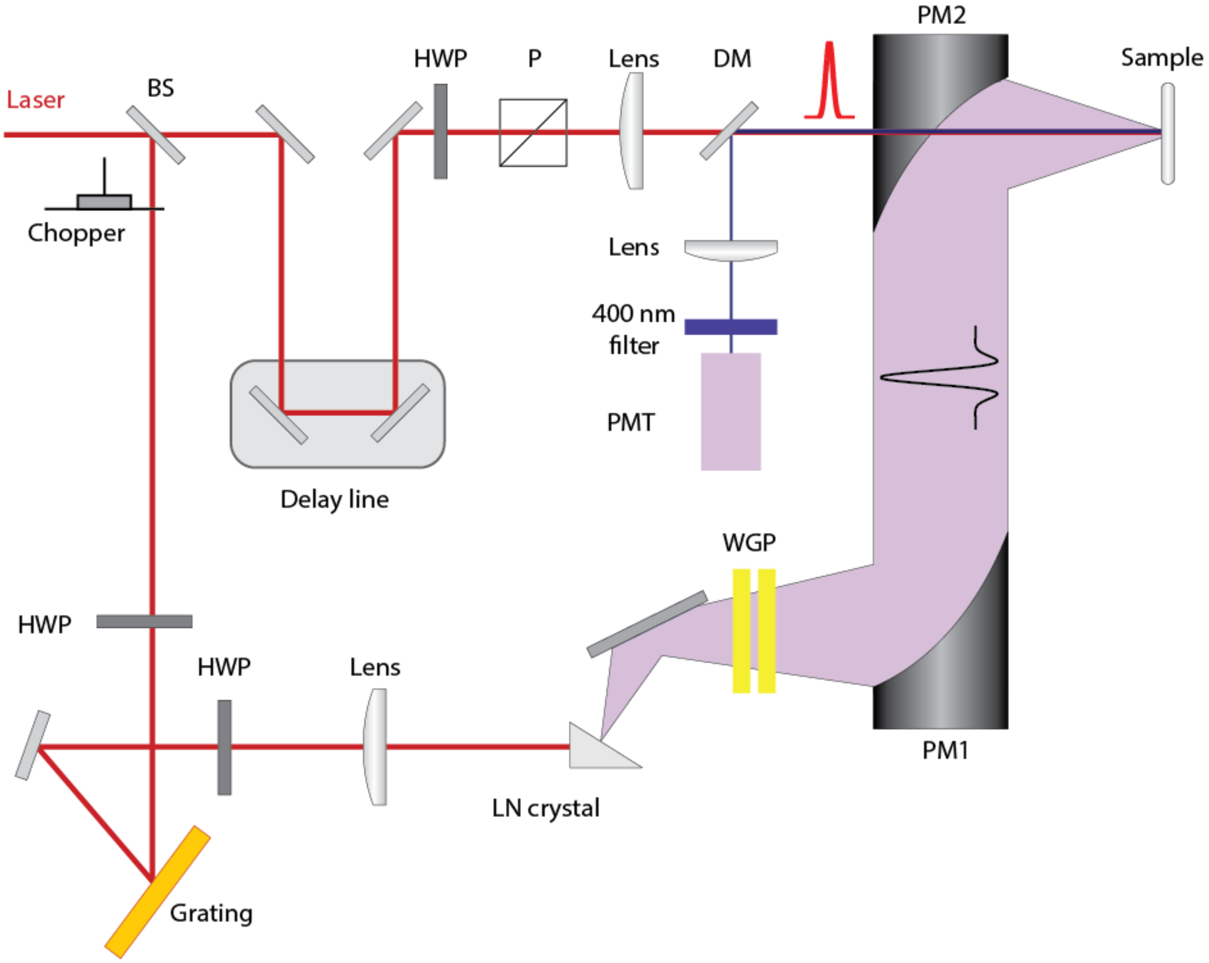

Fig. S5. Schematic illustration of the reflection TFISH experiment setup. BS: beam splitter; HWP: half-wave plate; P: polarizer; WGP: wire-grid polarizers; DM: dichroic mirror; PM: parabolic mirror; LN: lithium niobate crystal for THz generation; PMT: photomultiplier tube.

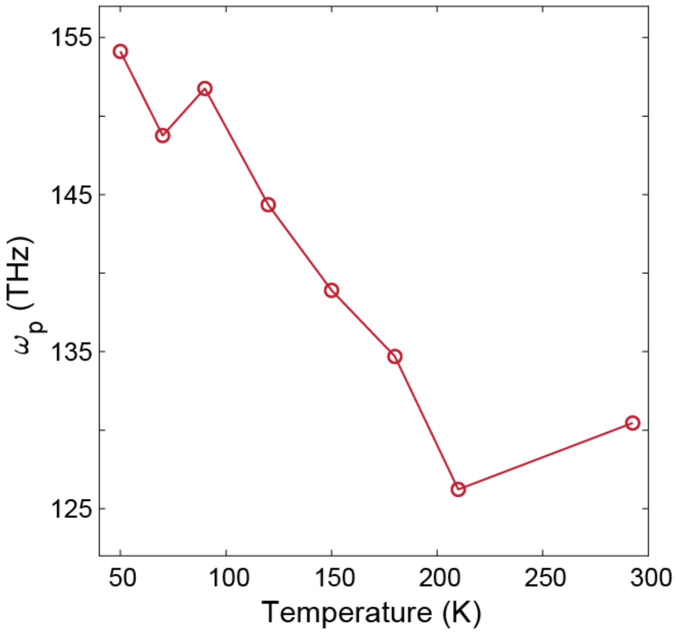

Fig. S6. Plasma frequency of PST-0.5. Temperature-dependent plasma frequency extracted from a fit of the time-domain THz spectroscopy data.

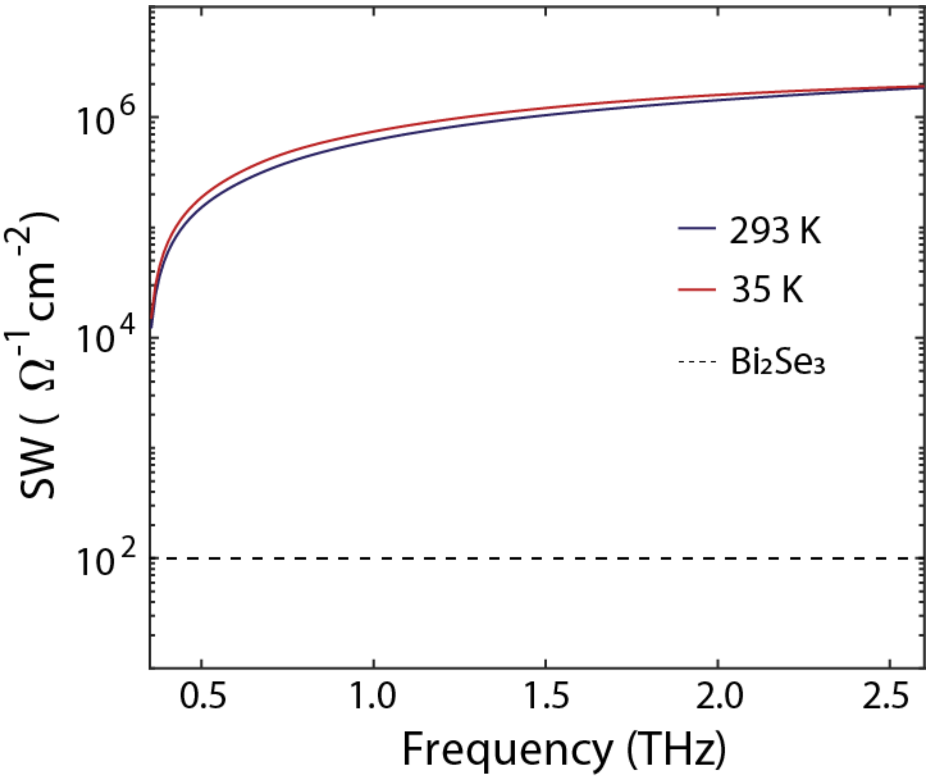

Fig. S7. THz spectral weight of PST-0.5 at representative temperatures. The dashed curve represents the contribution from the topological surface carriers as obtained from the data of Ref. (*7*).

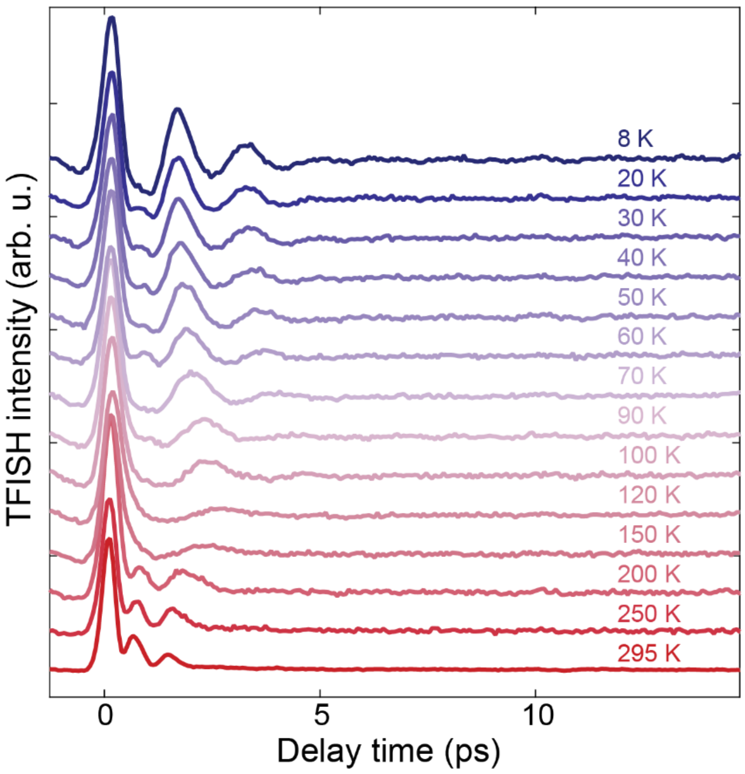

Fig. S8. Temperature dependent TFISH measurements for (111) PST-0.5 on a GaAs substrate on a finer temperature step size at low temperatures. Phonon mode softening and hardening can be observed from the TFISH signals and indicate the existence of an inversion symmetry-lifting phase transitions in the sample. The traces are vertically offset by a constant for clarity. The peak THz field strength used in the experiment is 630 kV/cm. In Fig. S10, we also show that the phonon frequencies exhibit no dependence on the THz field strength.

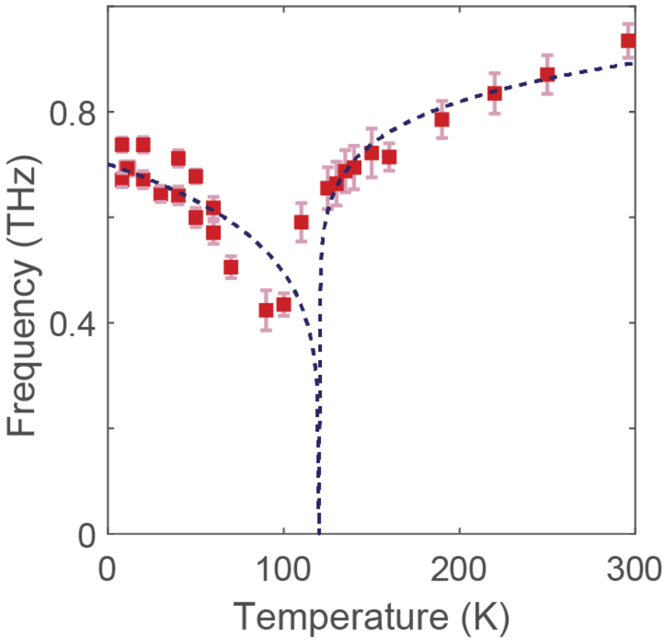

Fig. S9. Fit of the temperature-dependent mode frequency with Landau's theory. The low-temperature data has a critical exponent β = 0.19 ± 0.09. The high-temperature data has a critical exponent β = 0.11 ± 0.04. The fit follows $\boldsymbol{\omega_0 = \alpha|T - T_c|^{\beta}}$.

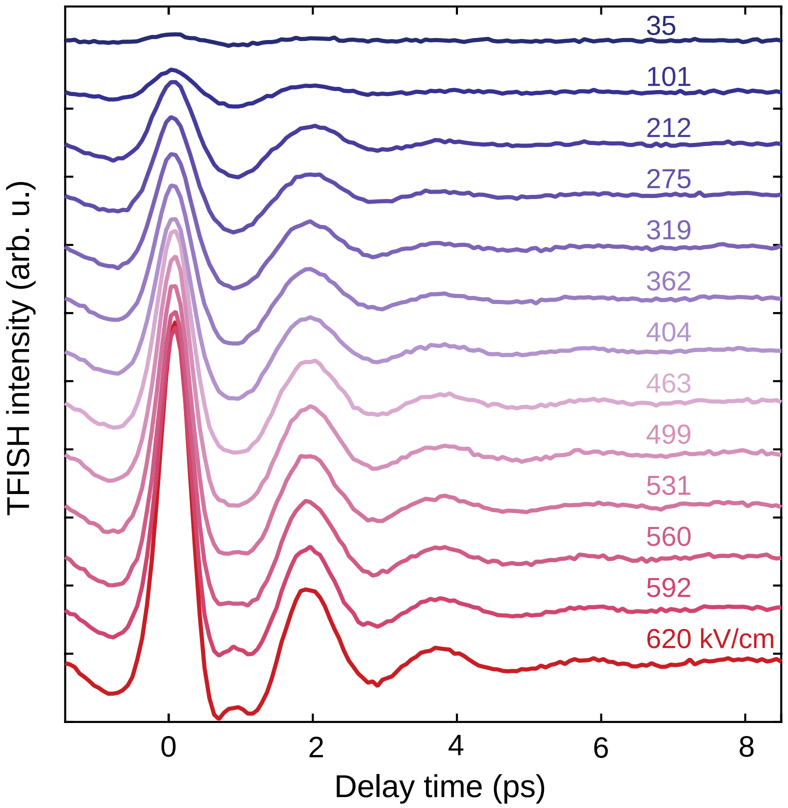

Fig. S10. TFISH field strength dependence on PST-0.5 at 60 K. TFISH time-domain signals under excitation with different THz field strengths. The traces are vertically offset by a constant step for clarity.

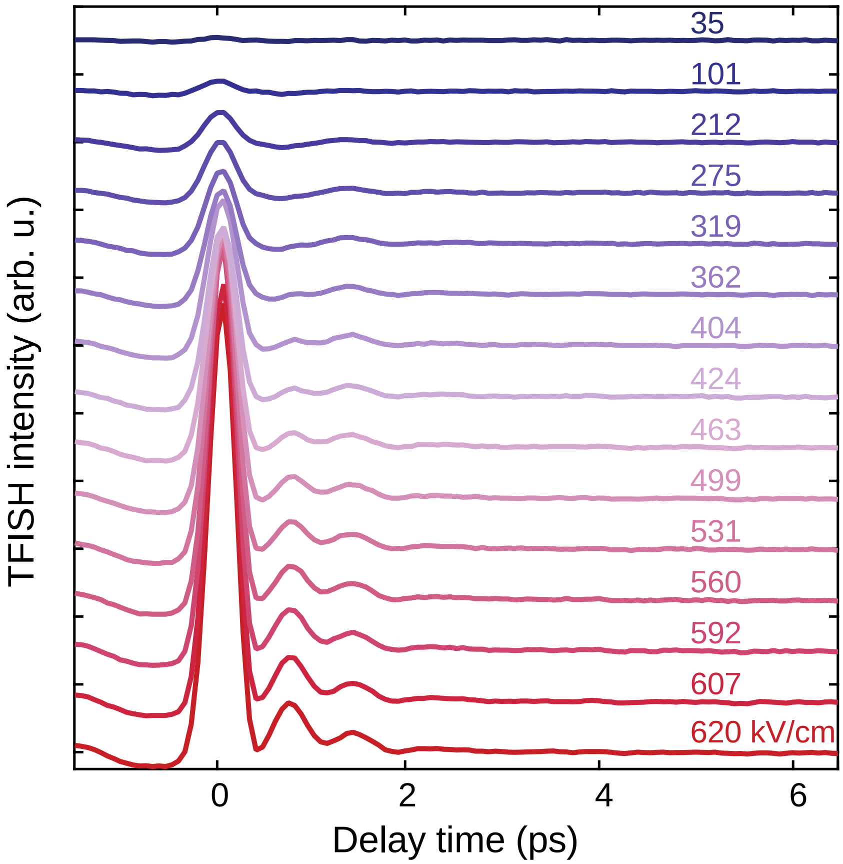

Fig. S11. TFISH field strength dependence on PST-0.5 at 295 K. TFISH time-domain signals under excitation with different THz field strengths. The traces are vertically offset by a constant step for clarity.

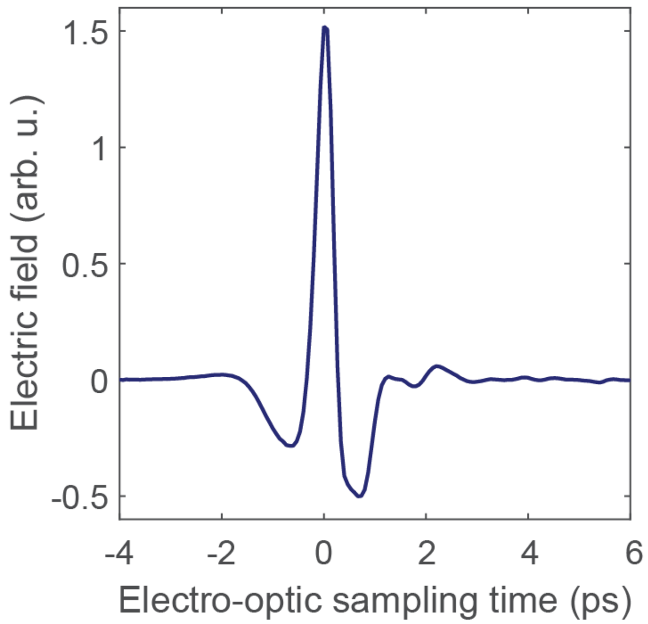

Fig. S12. The time-domain trace of the THz field used as a pump in the TFISH experiment. The trace was acquired via electro-optic sampling.

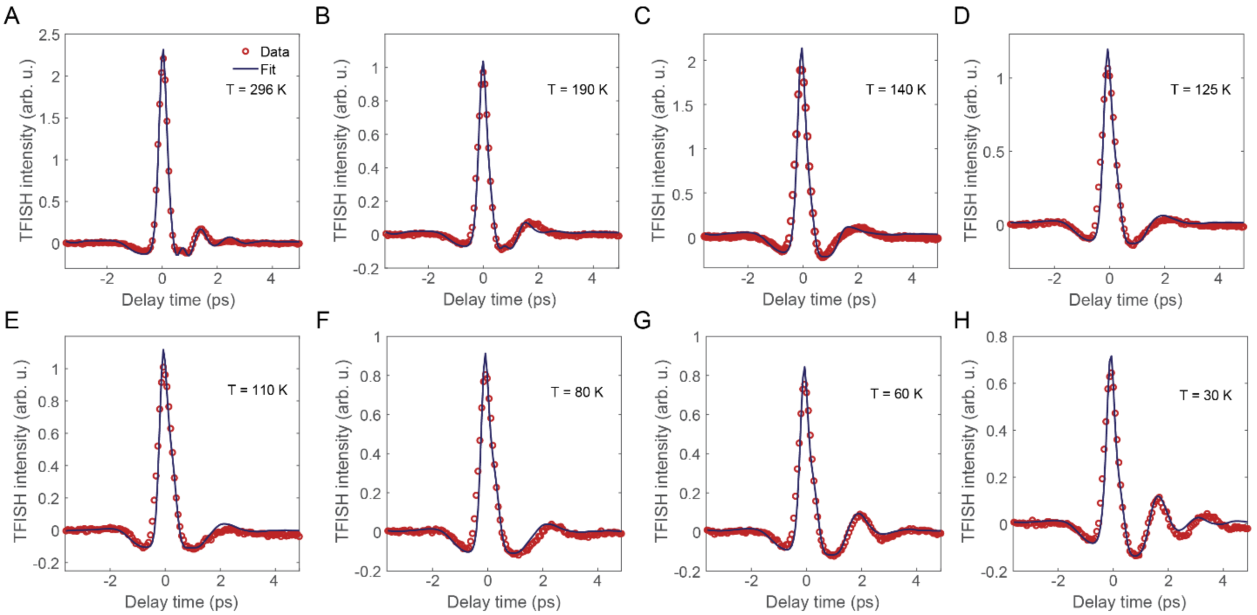

Fig. S13. Fits to the TFISH traces as a function of temperature for PST-0.5. (**A-H**) The three-component fit to the data in Fig. 2 of the main text at decreasing temperatures with the time-zero signal included. The extracted soft-mode frequencies are 0.93, 0.79, 0.69, 0.66, 0.59, 0.57, 0.62, and 0.69 THz. The extracted static values are 0.41, 0.31, 0.54, 0.37, 0.32, 0.35, 0.35, and 0.38 THz.

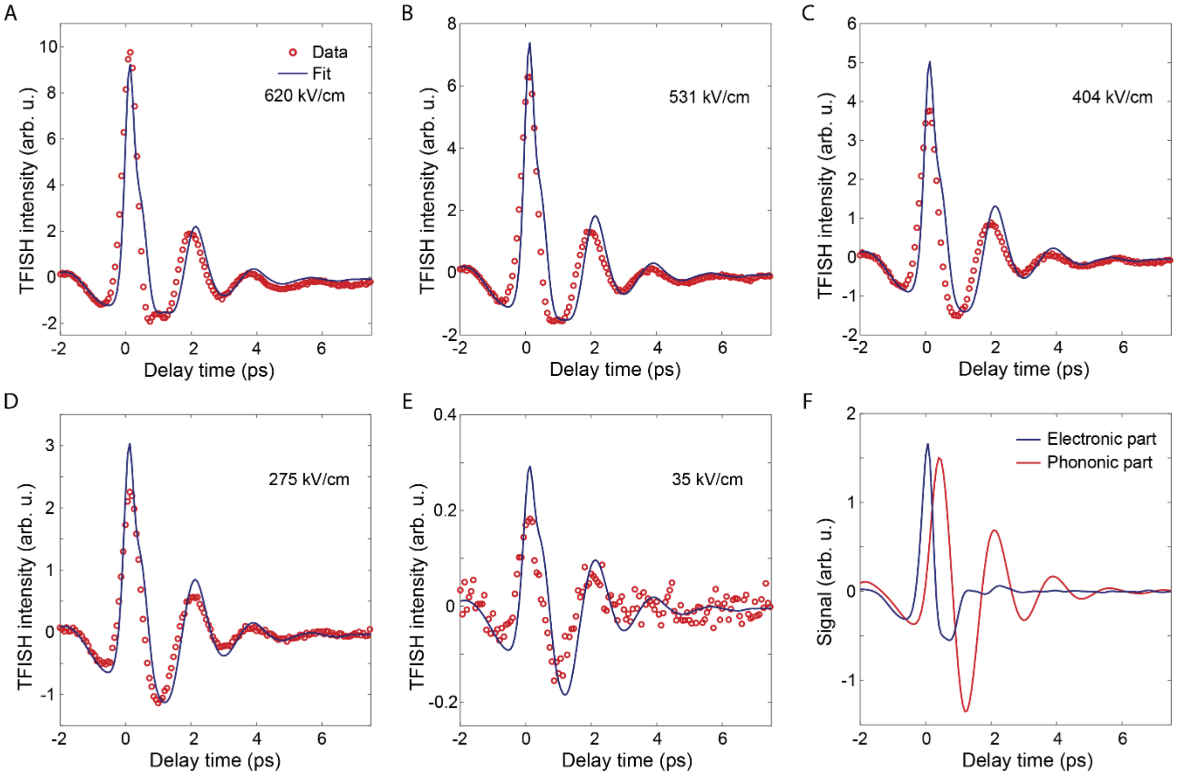

Fig. S14. **Fits to the TFISH traces as a function of THz field strength for PST-0.5 at 60 K.** (**A-E**) The three-component fit to the data in Fig. S10 at increasing THz field strengths with the time-zero signal included. (**F**) The relative amplitudes of the electronic and phononic responses extracted from the data taken at 620 kV/cm. The extracted soft-mode frequency is 0.58 THz; the damping rate is 0.20; the static amplitude is 1.2. They are fixed across all the traces at other field strengths.

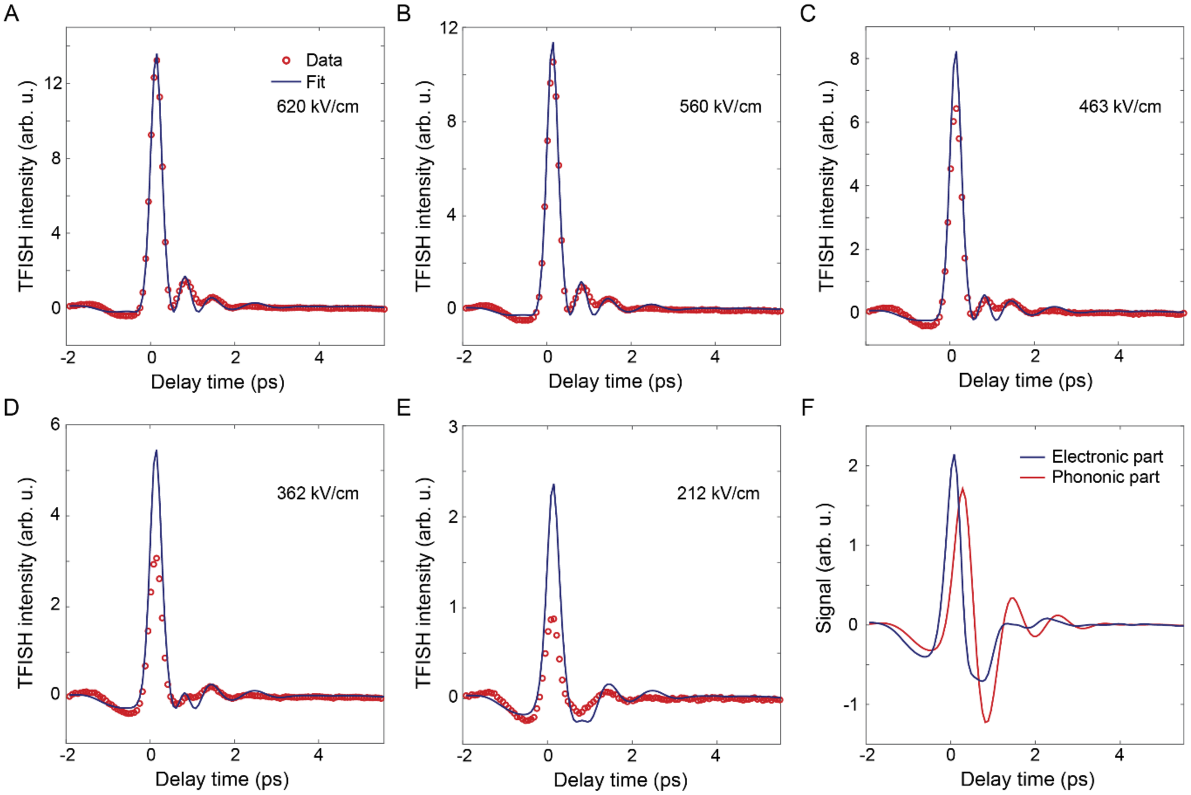

Fig. S15. **Fits to the TFISH traces as a function of THz field strength for PST-0.5 at 295 K.** (**A-E**) Fits to the data in Fig. S11 at variable THz field strengths based on electronic, phonon, and static contributions to the SHG signal intensity, as described in the text. **(F)** The electronic and phononic responses extracted from the data taken at 620 kV/cm, with the relative amplitudes used in the fit. The extracted soft-mode frequency is 1.0 THz; the damping rate is 0.31; the static amplitude is 0.53. They are fixed across all the traces at other field strengths.

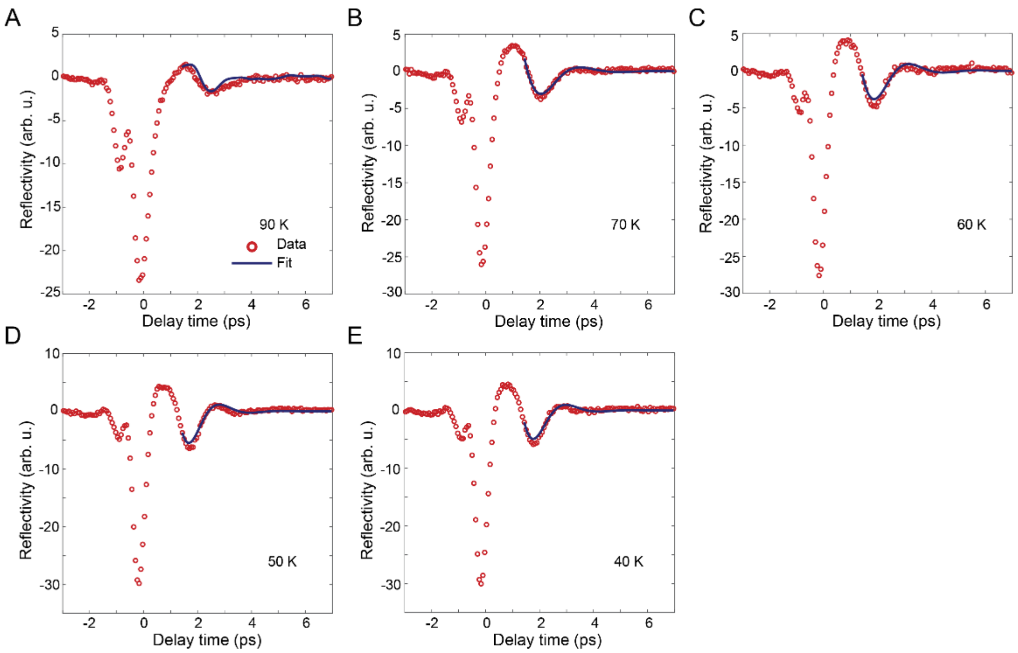

Fig. S16. Fits to the THz-field-induced transient reflectivity traces as a function of temperature for PST-0.5. The fit to the transient reflectivity data depicted in Fig. 4B of the main text was performed by using the numerical results obtained from the driven harmonic oscillator model.

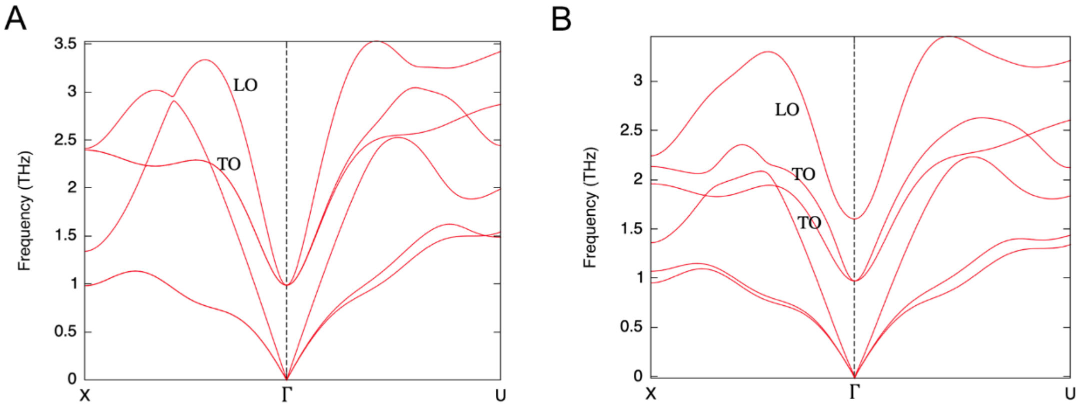

Fig. S17. The phonon spectrum of SnTe. (**A**) The phonon dispersion for SnTe without distortion. (**B**) The phonon spectra for SnTe with ferroelectric distortion.

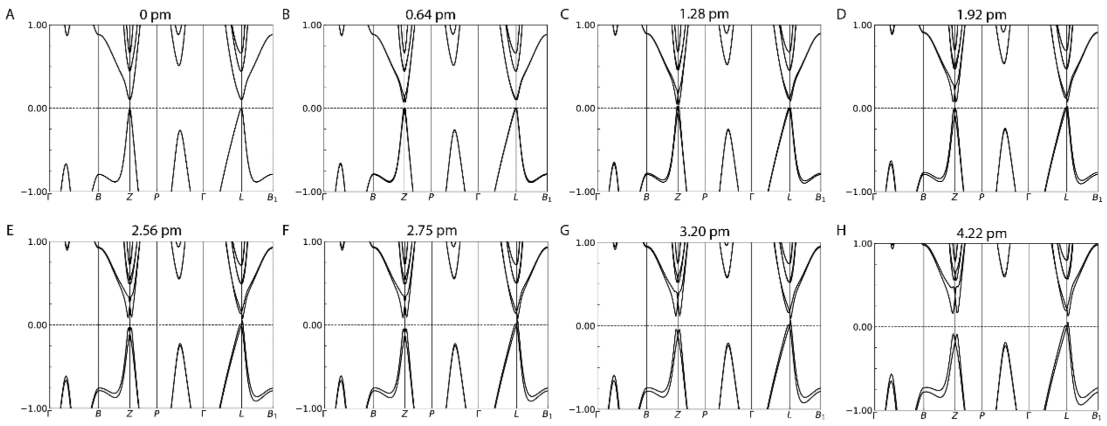

Fig. S18. Phonon-mediated topological phase transitions in SnTe. (A-H) Band structures of SnTe with varying distortions along the soft mode (from 0 pm to 4.22 pm).

	PST-0.2	PST-0.5	PST-0.7
T_{TCI}	NA	~ 110 K	Any
T_C	< 10 K	~ 120 K	~ 98 K

Table S1. Summary of the T_{TCI} and T_C in our samples.

REFERENCES AND NOTES

1. M. Dawber, K. M. Rabe, J. F. Scott, Physics of thin-film ferroelectric oxides. *Rev. Mod. Phys.* **77**, 1083–1130 (2005).

2. J. F. Scott, Applications of modern ferroelectrics. *Science* **315**, 954–959 (2007).

3. P. W. Anderson, E. I. Blount, Symmetry considerations on martensitic transformations: “Ferroelectric” metals? *Phys. Rev. Lett.* **14**, 217–219 (1965).

4. D. Hickox-Young, D. Puggioni, J. M. Rondinelli, Polar metals taxonomy for materials classification and discovery. *Phys. Rev. Mater.* **7**, 010301 (2023).

5. S. Bhowal, N. A. Spaldin, Polar metals: Principles and prospects. *Annu. Rev. Mat. Res.* **53**, 53–79 (2023).

6. N. A. Benedek, T. Birol, ‘Ferroelectric’ metals reexamined: Fundamental mechanisms and design considerations for new materials. *J. Mater. Chem. C* **4**, 4000–4015 (2016).

7. Y. Shi, Y. Guo, X. Wang, A. J. Princep, D. Khalyavin, P. Manuel, Y. Michiue, A. Sato, K. Tsuda, S. Yu, M. Arai, Y. Shirako, M. Akaogi, N. Wang, K. Yamaura, A. T. Boothroyd, A ferroelectric-like structural transition in a metal. *Nat. Mater.* **12**, 1024–1027 (2013).

8. N. J. Laurita, A. Ron, J.-Y. Shan, D. Puggioni, N. Z. Koocher, K. Yamaura, Y. Shi, J. M. Rondinelli, D. Hsieh, Evidence for the weakly coupled electron mechanism in an Anderson-Blount polar metal. *Nat. Commun.* **10**, 3217 (2019).

9. J. J. Gao, S.-Y. Fu, K. Yamaura, J. F. Lin, J.-S. Zhou, Room-temperature polar metal stabilized under high pressure. *Phys. Rev. B* **101**, 220101 (2020).

10. Z. Fei, W. Zhao, T. A. Palomaki, B. Sun, M. K. Miller, Z. Zhao, J. Yan, X. Xu, D. H. Cobden, Ferroelectric switching of a two-dimensional metal. *Nature* **560**, 336–339 (2018).

11. P. Sharma, F. Xiang, D.-F. Shao, D. Zhang, E. Y. Tsymbal, A. R. Hamilton, J. Seidel, A room-temperature ferroelectric semimetal. *Sci. Adv.* **5**, eaax5080 (2019).

12. Q. Yang, M. Wu, J. Li, Origin of two-dimensional vertical ferroelectricity in WTe_2 bilayer and multilayer. *J. Phys. Chem. Lett.* **9**, 7160–7164 (2018).

13. S. Lei, M. Gu, D. Puggioni, G. Stone, J. Peng, J. Ge, Y. Wang, B. Wang, Y. Yuan, K. Wang, Z. Mao, J. Rondinelli, V. Gopalan, Observation of quasi-two-dimensional polar domains and ferroelastic switching in a metal, $Ca_3Ru_2O_7$. *Nano Lett.* **18**, 3088–3095 (2018).

14. S. Lei, S. Chikara, D. Puggioni, J. Peng, M. Zhu, M. Gu, W. Zhao, Y. Wang, Y. Yuan, H. Akamatsu, M. H. W. Chan, X. Ke, Z. Mao, J. M. Rondinelli, M. Jaime, J. Singleton, F. Weickert, V. S. Zapf, V. Gopalan, Comprehensive magnetic phase diagrams of the polar metal $Ca_3(Ru_{0.95}Fe_{0.05})_2O_7$. *Phys. Rev. B* **99**, 224411 (2019).

15. D. Puggioni, M. Horio, J. Chang, J. M. Rondinelli, Cooperative interactions govern the fermiology of the polar metal $Ca_3Ru_2O_7$. *Phys. Rev. Res.* **2**, 023141 (2020).

16. Y. Yuan, P. Kissin, D. Puggioni, K. Cremin, S. Lei, Y. Wang, Z. Mao, J. M. Rondinelli, R. D. Averitt, V. Gopalan, Ultrafast quasiparticle dynamics in the correlated semimetal $Ca_3Ru_2O_7$. *Phys. Rev. B* **99**, 155111 (2019).

17. T. H. Hsieh, H. Lin, J. Liu, W. Duan, A. Bansil, L. Fu, Topological crystalline insulators in the SnTe material class. *Nat. Commun.* **3**, 982 (2012).

18. Y. Tanaka, Z. Ren, T. Sato, K. Nakayama, S. Souma, T. Takahashi, K. Segawa, Y. Ando, Experimental realization of a topological crystalline insulator in SnTe. *Nat. Phys.* **8**, 800–803 (2012).

19. D. Varjas, A. G. Grushin, R. Ilan, J. E. Moore, Dynamical piezoelectric and magnetopiezoelectric effects in polar metals from Berry phases and orbital moments. *Phys. Rev. Lett.* **117**, 257601 (2016).

20. L. Wu, S. Patankar, T. Morimoto, N. L. Nair, E. Thewalt, A. Little, J. G. Analytis, J. E. Moore, J. Orenstein, Giant anisotropic nonlinear optical response in transition metal monopnictide Weyl semimetals. *Nat. Phys.* **13**, 350–355 (2017).

21. H. Gao, Y. Kim, J. W. F. Venderbos, C. L. Kane, E. J. Mele, A. M. Rappe, W. Ren, Dirac-Weyl semimetal: Coexistence of Dirac and Weyl fermions in polar hexagonal *ABC* crystals. *Phys. Rev. Lett.* **121**, 106404 (2018).

22. X.-K. Wei, G. Bihlmayer, X. Zhou, W. Feng, Y. V. Kolen'ko, D. Xiong, L. Liu, S. Blügel, R. E. Dunin-Borkowski, Discovery of real-space topological ferroelectricity in metallic transition metal phosphides. *Adv. Mater.* **32**, e2003479 (2020).

23. C. W. Rischau, X. Lin, C. P. Grams, D. Finck, S. Harms, J. Engelmayer, T. Lorenz, Y. Gallais, B. Fauque, J. Hemberger, K. Behnia, A ferroelectric quantum phase transition inside the superconducting dome of $Sr_{1-x}Ca_xTiO_{3-\delta}$. *Nat. Phys.* **13**, 643–648 (2017).

24. A. Jindal, A. Saha, Z. Li, T. Taniguchi, K. Watanabe, J. C. Hone, T. Birol, R. M. Fernandes, C. R. Dean, A. N. Pasupathy, D. A. Rhodes, Coupled ferroelectricity and superconductivity in bilayer T_d-$MoTe_2$. *Nature* **613**, 48–52 (2023).

25. W. Luo, K. Xu, H. Xiang, Two-dimensional hyperferroelectric metals: A different route to ferromagnetic-ferroelectric multiferroics. *Phys. Rev. B* **96**, 235415 (2017).

26. H. Sakai, K. Ikeura, M. S. Bahramy, N. Ogawa, D. Hashizume, J. Fujioka, Y. Tokura, S. Ishiwata, Critical enhancement of thermopower in a chemically tuned polar semimetal $MoTe_2$. *Sci. Adv.* **2**, e1601378 (2016).

27. C. D. O'Neill, D. A. Sokolov, A. Hermann, A. Bossak, C. Stock, A. D. Huxley, Inelastic x-ray investigation of the ferroelectric transition in SnTe. *Phys. Rev. B* **95**, 144101 (2017).

28. E. Berger, S. Jamnuch, C. B. Uzundal, C. Woodahl, H. Padmanabhan, A. Amado, P. Manset, Y. Hirata, Y. Kubota, S. Owada, K. Tono, M. Yabashi, C. Wang, Y. Shi, V. Gopalan, C. P. Schwartz, W. S. Drisdell, I. Matsuda, J. W. Freeland, T. A. Pascal, M. Zuerch, Extreme ultraviolet second harmonic generation spectroscopy in a polar metal. *Nano Lett.* **21**, 6095–6101 (2021).

29. P. Dziawa, B. J. Kowalski, K. Dybko, R. Buczko, A. Szczerbakow, M. Szot, E. Łusakowska, T. Balasubramanian, B. M. Wojek, M. H. Berntsen, O. Tjernberg, T. Story, Topological crystalline insulator states in $Pb_{1-x}Sn_xSe$. *Nat. Mater.* **11**, 1023–1027 (2012).

30. V. V. Volobuev, P. S. Mandal, M. Galicka, O. Caha, J. Sánchez-Barriga, D. di Sante, A. Varykhalov, A. Khiar, S. Picozzi, G. Bauer, P. Kacman, R. Buczko, O. Rader, G. Springholz, Giant Rashba splitting in $Pb_{1-x}Sn_xTe$ (111) topological crystalline insulator films controlled by Bi doping in the bulk. *Adv. Mater.* **29**, 1604185 (2017).

31. Y. Okada, M. Serbyn, H. Lin, D. Walkup, W. Zhou, C. Dhital, M. Neupane, S. Xu, Y. J. Wang, R. Sankar, F. Chou, A. Bansil, M. Z. Hasan, S. D. Wilson, L. Fu, V. Madhavan, Observation of Dirac node formation and mass acquisition in a topological crystalline insulator. *Science* **341**, 1496–1499 (2013).

32. M. Serbyn, L. Fu, Symmetry breaking and Landau quantization in topological crystalline insulators. *Phys. Rev. B* **90**, 035402 (2014).

33. J. Liu, T. H. Hsieh, P. Wei, W. Duan, J. Moodera, L. Fu, Spin-filtered edge states with an electrically tunable gap in a two-dimensional topological crystalline insulator. *Nat. Mater.* **13**, 178–183 (2014).

34. I. Zeljkovic, Y. Okada, M. Serbyn, R. Sankar, D. Walkup, W. Zhou, J. Liu, G. Chang, Y. J. Wang, M. Z. Hasan, F. Chou, H. Lin, A. Bansil, L. Fu, V. Madhavan, Dirac mass generation from crystal symmetry breaking on the surfaces of topological crystalline insulators. *Nat. Mater.* **14**, 318–324 (2015).

35. M. Iizumi, Y. Hamaguchi, K. F. Komatsubara, Y. Kato, Phase transition in SnTe with low carrier concentration. *J. Physical Soc. Japan* **38**, 443–449 (1975).

36. Y. Okamura, H. Handa, R. Yoshimi, A. Tsukazaki, K. S. Takahashi, M. Kawasaki, Y. Tokura, Y. Takahashi, Terahertz lattice and charge dynamics in ferroelectric semiconductor $Sn_xPb_{1-x}Te$. *NPJ Quantum Mater.* **7**, 91 (2022).

37. F. G. G. Hernandez, A. Baydin, S. Chaudhary, F. Tay, I. Katayama, J. Takeda, H. Nojiri, A. K. Okazaki, P. H. O. Rappl, E. Abramof, M. Rodriguez-Vega, G. A. Fiete, J. Kono, Observation of interplay between phonon chirality and electronic band topology. *Sci. Adv.* **9**, eadj4074 (2023).

38. T. Shimada, K. L. I. Kobayashi, Y. Katayama, K. F. Komatsubara, Soft-phonon-induced Raman scattering in IV-VI compounds. *Phys. Rev. Lett.* **39**, 143–146 (1977).

39. W. J. Daughton, C. W. Tompson, E. Gurmen, Lattice instability and phonon lifetimes in $Pb_{1-x}Sn_xTe$ alloys. *J. Phys. C: Solid State Phys.* **11**, 1573–1581 (1978).

40. K.-P. Möllmann, K. H. Herrmann, R. Enderlein, Direct observation of ferroelectric phase in $Pb_{1-x}Sn_xTe$. *Phys. B+C* **117-118**, 582–584 (1983).

41. B. Cheng, P. Taylor, P. Folkes, C. Rong, N. P. Armitage, Magnetoterahertz response and Faraday rotation from massive Dirac fermions in the topological crystalline insulator $Pb_{0.5}Sn_{0.5}Te$. *Phys. Rev. Lett.* **122**, 097401 (2019).

42. K.-L. Yeh, M. Hoffmann, J. Hebling, K. A. Nelson, Generation of 10μJ ultrashort terahertz pulses by optical rectification. *Appl. Phys. Lett.* **90**, 171121 (2007).

43. D. Cook, J. Chen, E. Morlino, R. Hochstrasser, Terahertz-field-induced second-harmonic generation measurements of liquid dynamics. *Chem. Phys. Lett.* **309**, 221–228 (1999).

44. K. A. Müller, W. Berlinger, Static critical exponents at structural phase transitions. *Phys. Rev. Lett.* **26**, 13–16 (1971).

45. R. T. Bate, D. L. Carter, J. S. Wrobel, Paraelectric behavior of PbTe. *Phys. Rev. Lett.* **25**, 159–162 (1970).

46. M. P. Jiang, M. Trigo, I. Savić, S. Fahy, É. D. Murray, C. Bray, J. Clark, T. Henighan, M. Kozina, M. Chollet, J. M. Glownia, M. C. Hoffmann, D. Zhu, O. Delaire, A. F. May, B. C. Sales, A. M. Lindenberg, P. Zalden, T. Sato, R. Merlin, D. A. Reis, The origin of incipient ferroelectricity in lead telluride. *Nat. Commun.* **7**, 12291 (2016).

47. K. Ishioka, O. V. Misochko, in *Coherent Lattice Oscillations in Solids and Their Optical Control* (Springer Berlin Heidelberg, 2010), pp. 23–46.

48. T. P. Dougherty, G. P. Wiederrecht, K. A. Nelson, M. H. Garrett, H. P. Jensen, C. Warde, Femtosecond resolution of soft mode dynamics in structural phase transitions. *Science* **258**, 770–774 (1992).

49. T. Kohmoto, K. Tada, T. Moriyasu, Y. Fukuda, Observation of coherent phonons in strontium titanate: Structural phase transition and ultrafast dynamics of the soft modes. *Phys. Rev. B* **74**, 064303 (2006).

50. R. Lu, M. Hase, M. Kitajima, S. Nakashima, S. Sugai, Ultrafast critical dynamics of a ferroelectric phase transition in $Pb_{1-x}Ge_xTe$. *Phys. Rev. B* **75**, 012107 (2007).

51. Y. Koyama, T. Moriyasu, E. Okamura, Y. Yamada, K. Tanaka, T. Kohmoto, Doping-induced ferroelectric phase transition in strontium titanate: Observation of birefringence and coherent phonons under ultraviolet illumination. *Phys. Rev. B* **81**, 024104 (2010).

52. E. Baldini, C. A. Belvin, M. Rodriguez-Vega, I. O. Ozel, D. Legut, A. Kozlowski, A. M. Oles, K. Parlinski, P. Piekarz, J. Lorenzana, G. A. Fiete, N. Gedik, Discovery of the soft electronic modes of the trimeron order in magnetite. *Nat. Phys.* **16**, 541–545 (2020).

53. M. E. Ziffer, L. Huber, F. Wang, V. A. Posey, J. C. Russell, T. Handa, X. Roy, X.-Y. Zhu, Charge carrier coupling to the soft phonon mode in a ferroelectric semiconductor. *Phys. Rev. Mater.* **6**, 095401 (2022).

54. T. Nishijima, T. Watanabe, H. Sekiguchi, Y. Ando, E. Shigematsu, R. Ohshima, S. Kuroda, M. Shiraishi, Ferroic Berry curvature dipole in a topological crystalline insulator at room temperature. *Nano Lett.* **23**, 2247–2252 (2023).

55. S. R. Phillpot, V. Gopalan, Coupled displacive and order–disorder dynamics in $LiNbO_3$ by molecular-dynamics simulation. *Appl. Phys. Lett.* **84**, 1916–1918 (2004).

56. Q. Zhang, T. Cagin, W. A. Goddard III, The ferroelectric and cubic phases in $BaTiO_3$ ferroelectrics are also antiferroelectric. *Proc. Natl. Acad. Sci. U.S.A.* **103**, 14695–14700 (2006).

57. H. Holloway, J. Walpole, "MBE techniques for IV-VI optoelectronic devices" in *Molecular Beam Epitaxy*, Pamplin, B. R., Ed. (Pergamon, 1980), pp. 49–94.

58. T. C. Harman, P. J. Taylor, M. P. Walsh, B. E. LaForge, Quantum dot superlattice thermoelectric materials and devices. *Science* **297**, 2229–2232 (2002).

59. G. Kresse, J. Hafner, Ab initio molecular dynamics for liquid metals. *Phys. Rev. B* **47**, 558–561 (1993).

60. G. Kresse, J. Furthmüller, Efficient iterative schemes ab initio total-energy calculations using a plane-wave basis set. *Phys. Rev. B* **54**, 11169–11186 (1996).

61. A. Togo, I. Tanaka, First principles phonon calculations in materials science. *Scr. Mater.* **108**, 1–5 (2015).

62. R. E. Glover, M. Tinkham, Conductivity of superconducting films for photon energies between 0.3 and $40kT_c$. *Phys. Rev.* **108**, 243 (1957).

63. V. S. Kamboj, A. Singh, T. Ferrus, H. E. Beere, L. B. Duffy, T. Hesjedal, C. H. W. Barnes, D. A. Ritchie, Probing the topological surface state in Bi_2Se_3 thin films using temperature-dependent terahertz spectroscopy. *ACS Photonics* **4**, 2711–2718 (2017).

64. T. Liang, S. Kushwaha, J. Kim, Q. Gibson, J. Lin, N. Kioussis, R. J. Cava, N. P. Ong, A pressure-induced topological phase with large Berry curvature in $Pb_{1-x}Sn_xTe$. *Sci. Adv.* **3**, e1602510 (2017).

65. R. Valdés Aguilar, A. V. Stier, W. Liu, L. S. Bilbro, D. K. George, N. Bansal, L. Wu, J. Cerne, A. G. Markelz, S. Oh, N. P. Armitage, Terahertz response and colossal Kerr rotation from the surface states of the topological insulator Bi_2Se_3. *Phys. Rev. Lett.* **108**, 087403 (2012).

66. F. Giorgianni, E. Chiadroni, A. Rovere, M. Cestelli-Guidi, A. Perucchi, M. Bellaveglia, M. Castellano, D. di Giovenale, G. Di Pirro, M. Ferrario, R. Pompili, C. Vaccarezza, F. Villa, A. Cianchi, A. Mostacci, M. Petrarca, M. Brahlek, N. Koirala, S. Oh, S. Lupi, Strong nonlinear terahertz response induced by Dirac surface states in Bi_2Se_3 topological insulator. *Nat. Commun.* **7**, 11421 (2016).

67. X. Li, T. Qiu, J. Zhang, E. Baldini, J. Lu, A. M. Rappe, K. A. Nelson, Terahertz field-induced ferroelectricity in quantum paraelectric $SrTiO_3$. *Science* **364**, 1079–1082 (2019).

68. K. Koepernik, H. Eschrig, Full-potential nonorthogonal local-orbital minimum-basis band-structure scheme. *Phys. Rev. B* **59**, 1743 (1999).

69. Q. Xu, Y. Zhang, K. Koepernik, W. Shi, J. van den Brink, C. Felser, Y. Sun, Comprehensive scan for nonmagnetic Weyl semimetals with nonlinear optical response. *NPJ Comput. Mater.* **6**, 32 (2020).

70. Y. Wang, G. Luo, J. Liu, R. Sankar, N.-L. Wang, F. Chou, L. Fu, Z. Li, Observation of ultrahigh mobility surface states in a topological crystalline insulator by infrared spectroscopy. *Nat. Commun.* **8**, 366 (2017).

71. M. Kozina, M. Fechner, P. Marsik, T. van Driel, J. M. Glownia, C. Bernhard, M. Radovic, D. Zhu, S. Bonetti, U. Staub, M. C. Hoffmann, Terahertz-driven phonon upconversion in $SrTiO_3$. *Nat. Phys.* **15**, 387–392 (2019).

72. J. S. Cetnar, D. L. Rode, Fermi level and electrostatic screening factor in degenerate semiconductors and metal alloys. *J. Electr. Mater.* **48**, 3399–3404 (2019).

73. F. Weber, S. Rosenkranz, J.-P. Castellan, R. Osborn, G. Karapetrov, R. Hott, R. Heid, K.-P. Bohnen, A. Alatas, Electron-phonon coupling and the soft phonon modes in $TiSe_2$. *Phys. Rev. Lett.* **107**, 266401 (2011).

74. G. Grüner, The dynamics of charge-density waves. *Rev. Mod. Phys.* **60**, 1129 (1988).

75. C. Milesi-Brault, C. Toulouse, E. Constable, H. Aramberri, V. Simonet, S. de Brion, H. Berger, L. Paolasini, A. Bosak, J. Íñiguez, M. Guennou, Archetypal soft-mode-driven antipolar transition in francisite $Cu_3Bi(SeO_3)_2O_2Cl$. *Phys. Rev. Lett.* **124**, 097603 (2020).
